# Understanding *Crassostrea virginica* tolerance of *Perkinsus marinus* through global gene expression analysis

**DOI:** 10.3389/fgene.2023.1054558

**Published:** 2023-01-19

**Authors:** Dina A. Proestou, Mary E. Sullivan, Kathryn Markey Lundgren, Tal Ben-Horin, Erin M. Witkop, Keegan M. Hart

**Affiliations:** ^1^ National Cold Water Marine Aquaculture Center, USDA Agricultural Research Service, Kingston, RI, United States; ^2^ Department of Fisheries, Animal and Veterinary Science, University of Rhode Island, Kingston, RI, United States

**Keywords:** dermo, tolerance, eastern oyster, transcriptomics, WGCNA

## Abstract

Disease tolerance, a host’s ability to limit damage from a given parasite burden, is quantified by the relationship between pathogen load and host survival or reproduction. Dermo disease, caused by the protozoan parasite *P. marinus*, negatively impacts survival in both wild and cultured eastern oyster (*C. virginica*) populations. Resistance to *P. marinus* has been the focus of previous studies, but tolerance also has important consequences for disease management in cultured and wild populations. In this study we measured dermo tolerance and evaluated global expression patterns of two sensitive and two tolerant eastern oyster families experimentally challenged with distinct doses of *P. marinus* (0, 10^6^, 10^7^, and 10^8^ parasite spores per gram wet weight, *n* = 3–5 individuals per family per dose). Weighted Gene Correlation Network Analysis (WGCNA) identified several modules correlated with increasing parasite dose/infection intensity, as well as phenotype. Modules positively correlated with dose included transcripts and enriched GO terms related to hemocyte activation and cell cycle activity. Additionally, these modules included G-protein coupled receptor, toll-like receptor, and tumor necrosis factor pathways, which are important for immune effector molecule and apoptosis activation. Increased metabolic activity was also positively correlated with treatment. The module negatively correlated with infection intensity was enriched with GO terms associated with normal cellular activity and growth, indicating a trade-off with increased immune response. The module positively correlated with the tolerant phenotype was enriched for transcripts associated with “programmed cell death” and contained a large number of tripartite motif-containing proteins. Differential expression analysis was also performed on the 10^8^ dosed group using the most sensitive family as the comparison reference. Results were consistent with the network analysis, but signals for “programmed cell death” and serine protease inhibitors were stronger in one tolerant family than the other, suggesting that there are multiple avenues for disease tolerance. These results provide new insight for defining dermo response traits and have important implications for applying selective breeding for disease management.

## 1 Introduction

Commercially important bivalve species endure exposure to numerous viral, bacterial, and parasitic diseases from open-water environments, resulting in significant mass mortality and economic loss for the aquaculture industry (Zannella et al., 2017). For example, in 2008, juvenile Pacific oysters (*Crassostrea gigas*) in all French production areas began experiencing mass mortalities (40%–100%) linked to a new microvariant of Ostreid Herpesvirus type 1 (OsHV-1 µvar) (Segarra et al., 2010). Initial tissue damage by this more virulent strain, followed by opportunistic bacterial (*Vibrio splendidus* or *V. aestuarianus*) infections, causes Pacific Oyster Mortality Syndrome (POMS) which has spread to other locations in Europe, Australia, and New Zealand and is largely responsible for the dramatic reduction in global Pacific oyster production over the past 2 decades (Martenot et al., 2011; Keeling et al., 2014; Ugalde et al., 2018; Fuhrmann et al., 2019; Petton et al., 2021). In addition to POMS, members of the genus *Vibrio* (*V. tubiashii*, *V. coralliiyticus*) have been linked to episodes of near 100% mortality due to larval bacillary necrosis in multiple shellfish (*C. gigas*, *Ostrea edulis*, *Agropecten irradians*, *Perna canaliculus*) hatcheries (Travers et al., 2015). *V. tapetis* and *Roseovarius crassostreae*, which induce similar symptoms and rapid, large-scale mortalities in juvenile *Venerupis philippinarum* and eastern oysters (*C. virginica*), are responsible for Brown Ring Disease and Roseovarius Oyster Disease (Boettcher et al., 2000; Paillard, 2004). Conversely, protozoan parasites infiltrate all bivalve life stages, but negative impacts (primarily tissue wasting and death) typically manifest in adults. Bonamiosis, a disease caused by the haplosporidians *Bonamia ostreae* in the northern and *B. exitosa* in both northern and southern hemispheres, initiated the decline of European *O. edulis* in the 1970s and 2 decades later decimated the flat oyster population in New Zealand (Hine et al., 2001; Engelsma et al., 2014). *Marteilia* species, which compromise the digestive systems and lead to severe emaciation of *O. edulis*, *Cerastoderma edule*, *Mytilus galloprovincialis, Saccrostrea glomerata* and other bivalves in Europe and Australia have reduced production to less than 10% of historical values in several areas (Villalba et al., 2014; Carrasco et al., 2015).

Expanded distribution and heightened prevalence/intensity of many bivalve diseases over the last 40 years are partly a consequence of and threat to worldwide aquaculture production. International trade and new high-density rearing systems promote economic growth, but risk introduction and increased transmission of harmful microorganisms in new areas (Naylor et al., 2021). Most industry sectors have responded to the threat with best management practices and strict biosecurity protocols; however, these containment approaches are permeable (Carnegie et al., 2016). By exploiting standing genetic variation in bivalve species, selective breeding for resistance traits can augment existing disease control measures and industry profitability (Houston, 2017). Several field-performance trials, long-term monitoring programs, and heritability studies have demonstrated the potential for bivalve populations to respond favorably to persistent disease pressure. For example, *O. edulis* survivors of massive *Bonamia* outbreaks in Ireland tended to perform better than naïve populations at three parasite-endemic sites in Europe (Culloty et al., 2004). The negative impacts of MSX disease (which was detected in the late 1950s) on eastern oyster populations in the Chesapeake Bay, USA are waning despite continued high prevalence of the haplosporidian parasite in oyster tissues (Carnegie and Burreson, 2011). And resistance to OsHV-1 laboratory exposures was highly heritable (narrow sense heritability ranged from 0.4 to 0.99) at three different Pacific oyster life stages (Azéma et al., 2017).

For selection to be successful, well-defined disease response phenotypes are also needed. In general, the bivalve research and aquaculture community has used the term ‘resistance’ to refer to multiple, distinct mechanisms. Typically, reported disease resistance reflects population- or family-level survival in response to controlled or natural challenges, but survival measurements cannot differentiate modes of action by the host (Holbrook et al., 2021; Potts et al., 2021). Host response to disease can be characterized in terms of resistance and tolerance, which are not mutually exclusive. Resistance is defined as the ability of a host to minimize infection by slowing pathogen proliferation and/or eliminating the pathogen altogether. It can be quantified as the inverse of pathogen load or change in pathogen load over time, but it is important to note that high resistance may not equate to high survival if there is a significant cost to pathogen elimination (Råberg et al., 2007; Råberg et al., 2009). Avoidance can also contribute to resistance when hosts prevent pathogen entry. Tolerant phenotypes display uncompromised fitness (survival, reproduction) below some threshold infection level (Råberg et al., 2007; Lefèvre et al., 2011) and can be assessed by evaluating performance in a range of pathogen concentrations. Because resistance and tolerance represent different ways of coping with pathogens, they can be treated as unique targets for selection.

Global transcriptome profiling has proven very useful in understanding bivalve-pathogen interactions (Nguyen and Alfaro, 2020) and provides an opportunity to further refine disease response phenotypes by comparing gene expression patterns in control and exposed or resistant/tolerant and sensitive groups. By assessing gene expression in infected Manilla clam extrapallial fluids compared to control, Rahmani et al. (2019) observed that *V. tapetis* effected changes in clam cell morphology and lysozyme activity that significantly reduced phagocytic capacity. Immediate upregulation of genes involved in pattern recognition, immune signaling, extracellular matrix remodeling, regulation of reactive oxygen species, and protease inhibition was observed in *O. edulis* populations with a long history of disease relative to naïve populations after challenge, suggesting rapid immune response is a key to resistance (Ronza et al., 2018). Basal transcriptome (in the absence of disease challenge) comparisons between multiple POMS-resistant *C. gigas* families and their sensitive counterparts also highlighted the importance of elevated pathogen recognition, anti-viral signaling, stress response, protein modification, and DNA repair in the resistant phenotype (de Lorgeril et al., 2020).

The protozoan parasite *P. marinus* is the primary cause of mortality at most eastern oyster culture sites throughout the Western Atlantic and Gulf of Mexico, United States. It causes dermo disease, a chronic condition characterized by inflammation, digestive epithelial tissue necrosis, and pallial tissue edema in adult individuals (Smolowitz, 2013). Like *Bonamia* species, *P. marinus* infects oyster blood cells (hemocytes) and multiplies within them. It can evade hemocyte-specific immune responses post-phagocytosis until they burst and release new *P. marinus* trophozoites, facilitating infection spread within the organism (Villalba et al., 2004). While genetic parameters for dermo resistance have not been quantified (Dégremont et al., 2015), results from several field performance trials suggest survival in disease-endemic areas is a heritable trait that varies among oyster lines historically exposed to divergent disease pressures (Ragone Calvo et al., 2003; Encomio et al., 2005; Frank-Lawale et al., 2014; Proestou et al., 2016). Additional dermo-response phenotypes have been described and measured using laboratory experiments. Significant differences in parasite avoidance behavior (particle clearance rate and time spent feeding in the presence of *P. marinus*) were detected among genetically distinct groups of eastern oysters (Ben-Horin et al., 2018a). A 6-week laboratory dermo challenge that tracked survival and parasite concentrations in eastern oyster tissues over time also identified differences in resistance (measured as the slope of the line depicting the relationship between parasite load and time) among multiple families (Proestou et al., 2019). Furthermore, differential gene expression analysis of *P. marinus*-injected and uninfected control oysters from one resistant and one sensitive eastern oyster family indicated that resistant oysters mounted an immediate response to the parasite that involved regulation of proteolysis, peptidase inhibitor activity, and oxidoreductase activity. Dermo-sensitive oysters exhibited a delayed, disorganized response to the parasite (Proestou and Sullivan, 2020).

Understanding and distinguishing among dermo response phenotypes and the genomic mechanisms that drive them has important breeding and ecological implications. By definition, resistance reduces pathogen fitness, thereby promoting pathogen evolution and a cyclical evolutionary arms race. Because tolerance has a neutral or positive effect on pathogens, there is no pressure on the host and pathogen to continuously coevolve (Råberg et al., 2009). A recent study modeling the impact of oyster aquaculture on sympatric wild populations suggests that tolerant phenotypes in culture can reduce environmental parasite densities and disease-related mortality in the wild (Ben-Horin et al., 2018b). Thus, tolerance rather than resistance may be a better selection target for managing dermo disease in aquaculture settings and preserving the health of wild eastern oyster reefs nearby. Here we build on previous work that measured the fitness (survival) of several distinct genetic groups of eastern oysters exposed to multiple *P. marinus* concentrations *via* injection in the adductor muscle (Peterson et al., 2020). Global gene expression patterns from four eastern oyster families (two at each end of the survival spectrum) were analyzed to assess the effect of dose on oyster response to the parasite and identify transcripts/gene networks/gene ontologies that discriminate tolerant from sensitive oysters.

## 2 Materials and methods

### 2.1 Oysters

In the summer of 2018, 1-year old seed oysters (20–64 mm shell height) from the Aquaculture Genetics and Breeding Technology Center (ABC) eastern oyster breeding program at the Virginia Institute of Marine Science (VIMS) were received by the USDA ARS NCWMAC and challenged with multiple doses (0, 10^6^, 10^7^, and 10^8^ cells g^−1^ tissue wet weight) of cultured, log-phase *P. marinus* (ATCC 50509, “DBNJ”) in the laboratory as described in Peterson et al., 2020. Prior to challenge, twelve pedigreed, full-sib families (products of single pair spawns) were sampled and tissues preserved in 95% ethanol (*n* = 10 per family) to assess levels of dermo infection from the field. During the laboratory experiment, *n* = 50 oysters per family, were injected with each dose and phenotyped for survival over the course of 63 days. Ten animals per family, per dose were phenotyped for parasite load at day 7 and day 50 post-exposure. Families were also phenotyped for parasite elimination/proliferation rate at each dose. Based on the disease challenge results, four families that represented the range of the phenotypic spectrum were chosen for global gene expression analysis.

### 2.2 Sample preparation

At 7 and 50 days post-injection, three 0-dose oysters and ten 10^6^, 10^7^, and 10^8^- dose oysters from each family were censored from the experiment and mantle tissue was preserved in RNA later. DNA was extracted from approximately 10 mg preserved mantle from each sampled individual with the Kurabo QuickGene DNA tissue kit (fk-dts; Autogen Inc.) and digested with RNase A for 2 min to remove co-purified RNA. DNA quantity and quality were assessed on a nanodrop spectrophotometer and extracts were subsequently diluted to a standard 100 ng ul^−1^ concentration. To estimate infection levels in each sampled oyster, 1 µL diluted DNA was added to a *P. marinus*-specific quantitative polymerase chain reaction (qPCR) assay (De Faveri et al., 2009). Parasite load was initially measured as the number of *P. marinus* DNA copies in 100 ng of total DNA and subsequently converted to (log) spores g^−1^ wet tissue weight as described in Proestou et al. (2019). Mantle tissue is recommended when using PCR-based methods to diagnose and quantify the intensity of dermo disease by the World Organization of Animal Health (2022).

Total RNA was isolated from a subset (3–5) of oysters from each family and dose that were censored on Day 7. Preserved mantle tissue (10 mg) was homogenized in 750ul TRI Reagent^®^ (MilliporeSigma, Burlington MA, United States) with an Omni Tip homogenizer (Omni International, Kennesaw GA, United States) and RNA was extracted following the manufacturers’ instructions. Extract yields were measured on a NanoDrop^™^ 8,000 spectrophotometer while quality assessments were obtained with Qubit^™^ RNA IQ Assay Kit (Thermo Scientific, Waltham MA, United States), which calculates an integrity and quality (IQ) number based on the percentage of large and small (degraded) RNA molecules present in a sample. 2.5 µg aliquots of high-quality RNA extracts (IQ > 6.2; mean IQ = 8.3) were treated with DNA-free^™^ DNase (Invitrogen, Waltham MA, United States) to remove DNA contamination and re-quantified with both the NanoDrop^™^ 8,000 and the Qubit^™^ RNA HS Assay Kit. We chose to extract RNA from and focus our expression analysis on mantle tissue because oyster immune effector cells (hemocytes) are well distributed throughout the mantle tissue (Allam and Raftos, 2015) and it is difficult to recover sufficient yields of high-quality RNA from hemolymph collected from individual 1-year old seed oysters.

### 2.3 Library construction and sequencing

Individually tagged sequencing libraries were prepared according to the TruSeq Stranded mRNA (Illumina, San Diego CA, United States) protocol. Approximately 1 μg DNase-treated total RNA was added to each preparation and 300–350 base pair (bp) mRNA fragments were purified, converted to cDNA, ligated to adaptors and enriched. Library quantity, quality, and insert size were evaluated using the Qubit™ DNA BR Assay Kit, and the Agilent High Sensitivity DNA chip kit with the Agilent 2,100 Bioanalyzer Instrument System. Equimolar amounts of high-quality libraries (>20 nM concentration) were subsequently combined into pools such that biological replicates were evenly distributed across pools. Quality control of library pools was confirmed with the KAPA Library Quant Kit (Illumina) LightCycler^®^ 480 qPCR Mix on the Roche LightCycler^®^ 96 qPCR machine before submission to Admera Health Biopharma Services (New Jersey, USA) for sequencing. Pools were paired-end sequenced on eight lanes of the Illumina HiSeq X Ten sequencing platform using the standard 2 × 150 kit. Sequence data were de-multiplexed and checked for quality with FastQC by the service provider. An additional data trimming step to remove low quality bases (<Q20) and fragments <50bp was performed in CLC Genomics Workbench V21 (https://www.qiagenbioinformatics.com/) using the Trim Reads tool.

Apart from the length fraction setting (which we reduced to 0.6 to account for high polymorphism in oysters (Zhang L. et al., 2014), we used the default settings in the CLC Genomics Workbench RNA-Seq Analysis tool to map trimmed reads to the *C. virginica* genome v 3.0 (NCBI; GCA_002022765.4) and generate a gene expression profile for each sequenced oyster. To avoid mapping to spurious assembly artifacts, a haplotig masked version of the genome was used as the reference (Puritz et al., 2022). Reads that mapped to >10 locations or in broken pairs were not counted to minimize inaccuracies in expression signals (Katz et al., 2010; Anders et al., 2013).

### 2.4 Phenotype analysis

Family-level responses to *P. marinus* infection were measured within and across doses to characterize dermo tolerant and resistant phenotypes. Survival probabilities, stratified by dose, were calculated for each family using non-parametric Kaplan-Meier estimation and Cox proportional hazards models were applied to detect significant differences between doses. All survival analyses were performed with the Survival v 3.2–13 package in R. Parasite load ((log) *P. marinus* spores g^−1^ wet tissue weight) data at 7 days post-injection were summarized for each group to confirm that infection intensities increased with dose and doses were consistent across families. Parasite elimination/proliferation rates were calculated as the change in parasite load between day 7 and day 50 post-injection using linear regression and slopes of regressions were compared to identify differences in resistance among groups.

### 2.5 Global expression analysis

We restricted our RNAseq and expression analyses to individuals censored at Day 7 post-injection. These oysters were the focus because previous work identified peak infection intensities and significant transcriptomic responses to the parasite at this time point (Proestou et al., 2019; Proestou and Sullivan, 2020). Specific animals were selected based on the median (log) spores g^−1^ wet weight for each group to minimize within group variation in transcriptomic response to the parasite. Overall expression patterns and their relationship to family and dose variables were visualized for all sequenced individuals by performing a principal component analysis (PCA) using the R function prcomp on transformed total read counts. Transformation to reduce overfitting was performed using the vst function in the R package DESeq2 v 1.32.0 (Love et al., 2014) with the option blind = FALSE and DESeq2 dataset design = ∼ Family_Dose (Supplementary Material S1).

To gain a more comprehensive understanding of the associations among expressed genes in our dataset and how clusters (modules) of similarly expressed transcripts correlate with external variables/traits of interest, we ran a Weighted Gene Correlation Network Analysis (WGCNA) with all sequenced samples simultaneously using the R package WGCNA v 1.70–3 (Langfelder and Horvath, 2008). Only transcripts with counts ≥10 across samples within at least one family (46,734) were included. Filtered transcripts were subject to variance stabilizing transformation using the vst function as described above. We used a soft threshold power of 8 (to satisfy scale-free topology), signed TOM type, and recommended default values for all other parameters to construct signed networks. The cut-off used to merge highly correlated modules was calculated based on sample size using the dynamicMergeCut function. Module eigengenes were correlated to traits of interest. Traits were coded for each family (1 or 0), treatment (1 = field + lab infected, 0 = field infected), dose (0 = field infected, 1 = 10^6^, 2 = 10^7^, 3 = 10^8^), sensitive phenotype (1 or 0), tolerant phenotype (1 or 0), and (log) spores g^−1^ wet weight (continuous variable). Enrichment analysis was used to explore modules highly and significantly correlated (*r* ^2^ ≥ 0.40 and *p*-value ≤0.001) with family and dose further (Supplementary Material S1).

A differential expression analysis was also conducted using the R package DESeq2 (Love et al., 2014) to isolate specific genes and gene ontologies whose expression may dictate dermo tolerance. Because our PCA and WGCNA analyses showed clear effects of family/phenotype and treatment on gene expression patterns and the 10^8^ dose best discriminated phenotypes, we performed pairwise comparisons between the most sensitive family and the other three families in this study with 10^8^ samples only. For this analysis, genes with fewer than 10 transcript counts across 10^8^ samples were removed (43,867 remained) and dataset design = ∼ family was used, specifying “90” as the reference level. Results were called for each coefficient (“family_84_vs._90”, “family_89_vs._90”, and “family_120_vs._90”) and approximate posterior estimation for a generalized linear model (apeglm; Zhu et al., 2019) shrinkage was applied to Log2 fold change (L2FC) estimates to reduce variance from low count noise without over shrinking (Zhu et al., 2019). Transcripts with a Benjamini Hochberg’s adjusted *p*-value (to control for false discovery rate) ≤ 0.05 were considered differentially expressed and subject to functional enrichment analysis. Shared differentially expressed transcripts (DET) among comparisons were identified using the R package VennDiagram v 1.7.3 (Supplementary Material S1).

For functional enrichment of interesting modules from the WGCNA analysis and DETs from the DESeq2 analysis, we used adaptive clustering combined with a Mann-Whitney U (MWU) test (Wright et al., 2015; https://github.com/z0on/GO_MWU). To assess gene ontology (GO) term enrichment, GO terms were first mapped to *C. virginica* transcripts and evaluated for annotation quality using OmicsBox v. 1.3.3 (Götz et al., 2008) as described in Proestou and Sullivan (2020). For module enrichment, we used signed WGCNA module analysis mode because it incorporates the eigengene-based module membership score (kME) of each transcript assigned to a module for additional power. This mode runs a global Fisher’s exact test for presence-absence of GO terms followed by a within-module MWU test for each GO category (Biological Process, BP; Cellular Component, CC; and Molecular Function, MF) where the table of measure for each module consists of the kME value for transcripts belonging to that module and a 0 value for transcripts included in the WGCNA but not assigned to that module, restricting the reference transcript set to transcripts included in WGCNA. Differentially expressed transcripts identified in the DESeq2 analysis were tested for enrichment in standard mode using the continuous value characterization strategy similar to that used in de Lorgeril (2020), which ranks each significant differentially expressed transcript by their L2FC value and assigns a value of 0 to all other transcripts included in the DESeq2 analysis to account for both L2FC expression values and significance levels while restricting the reference transcript set to include only transcripts included in the DESeq2 analysis. Enrichment results (FDR-corrected significant GO categories and corresponding thresholds) for both WGCNA modules and DETs were displayed as hierarchical clustering trees. Thresholds were adjusted for trees with a very large number of GO terms to assist visualization. Aside from the above-mentioned details, default parameters were used (Supplementary Material S1).

## 3 Results

### 3.1 Dermo response phenotypes in oyster families

**FIGURE 1:**
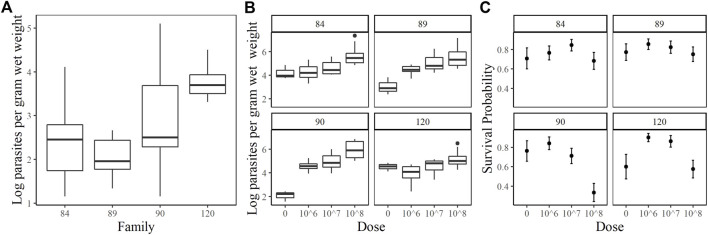
Oyster *P. marinus* response phenotypes. **(A)** Field infection intensities for each family prior to experiment start. **(B)** Parasite load measured at Day 7 post-injection in censored oysters. **(C)** Survival probabilities ± standard error for each family at each dose.

The families included in this study were 2017084 (84), 2017089 (89), 2017090 (90) and 2017120 (120). They were chosen based on their phenotypic response to increasing doses of *P. marinus*. Unfortunately, oysters arrived at the USDA ARS NCWMAC with field infections that varied in intensity within and among families (Figure 1A). Despite the high prevalence of natural infections in our experimental animals, by and large, pre-existing parasite loads were very light (≤1,000 spores g^−1^ wet weight; Johnson et al., 2020) or light (1,001–10,000 spores g^−1^ wet weight; (La Peyre et al., 2018). Parasite load did not change significantly over time in the 0 dose oysters, except for family 90 where mean log spores g^−1^ wet weight increased by two orders of magnitude (data not shown). On day 7 post-injection, measured parasite loads increased with dose in each family, although mean parasite load did not differ significantly between the 10^6^ and 10^7^ dose in any family (Figure 1B). Mean day 7 parasite loads in the 10^8^ dose were the highest and consistent among families, ranging from 5.18–5.97 (log) spores g^−1^ wet weight. Parasite elimination/proliferation rates (the slope of the relationship between (log) spores g^−1^ wet weight and time) were not significantly different from zero for most family/dose combinations aside from family 90 at dose 0 (positive slope), family 84 at dose 10^8^ and family 120 at the 10^7^ dose (slightly negative slopes). The percent mortality across all families and doses included in this experiment was 23.44%; however, survival patterns within families with respect to dose varied considerably (Figure 1C). At the end of the experiment, survival probabilities exceeded 0.70 in families 84 and 89 and no significant dose effect was detected (*p* = 0.6322 and 0.6581 for family 84 and 89 respectively). Survival probabilities were lower, particularly at the 10^8^ dose, in families 90 (0.33) and 120 (0.58). For these families, oysters in the 10^8^ dose were 3.5–7.6 times more likely to die than those in the lower doses. Based on our phenotypic measurements, we classified families 90 and 120 as sensitive and families 84 and 89 as tolerant to *P. marinus*.

### 3.2 Sequencing, read mapping, and overall expression patterns

A total of 5,417,510,884 high quality (sample average of 98% Q > 30) paired reads were sequenced from 61 animals (sample average of 88,811,654), with 96% of reads remaining after trimming (sample average of 85,636,140; Table 1). Across all samples, an average of 93.2% of paired reads mapped to the masked genome, 2.8% mapped in broken pairs and 4% of pairs were not mapped. To increase confidence in read assignment, broken pairs were not counted in downstream analyses (Katz et al., 2010).

**TABLE 1:** Sequencing and mapping statistics by group.

Family	Dose	Number of samples	Average raw read count	Average trimmed read count	% pairs mapped	% broken pairs mapped (not counted)	% pairs not mapped (not counted)
84	10^6	4	67,641,066	65,107,085	94.7	1.9	3.4
84	10^7	4	90,125,628	86,543,181	92.1	4.4	3.5
84	10^8	4	89,479,576	86,827,306	94.2	2.3	3.5
84	Control	3	107,668,881	102,538,733	92.9	3.4	3.7
89	10^6	4	75,255,607	73,093,650	94.1	2.5	3.4
89	10^7	5	103,112,262	99,411,905	94.0	2.7	3.3
89	10^8	4	75,369,488	72,692,425	93.8	2.5	3.7
89	Control	3	87,348,083	84,331,401	93.5	3.1	3.4
90	10^6	4	63,779,434	61,271,354	88.9	2.3	8.7
90	10^7	4	81,058,872	78,411,030	94.3	2.2	3.5
90	10^8	4	75,494,904	73,203,706	93.7	2.4	3.9
90	Control	3	125,657,239	120,745,181	92.9	3.6	3.5
120	10^6	4	108,160,734	103,890,656	93.7	2.6	3.8
120	10^7	4	88,900,404	85,872,472	93.8	2.5	3.7
120	10^8	4	86,514,950	83,822,642	92.6	3.0	4.3
120	Control	3	110,934,771	106,985,678	92.8	3.4	3.8

**FIGURE 2:**
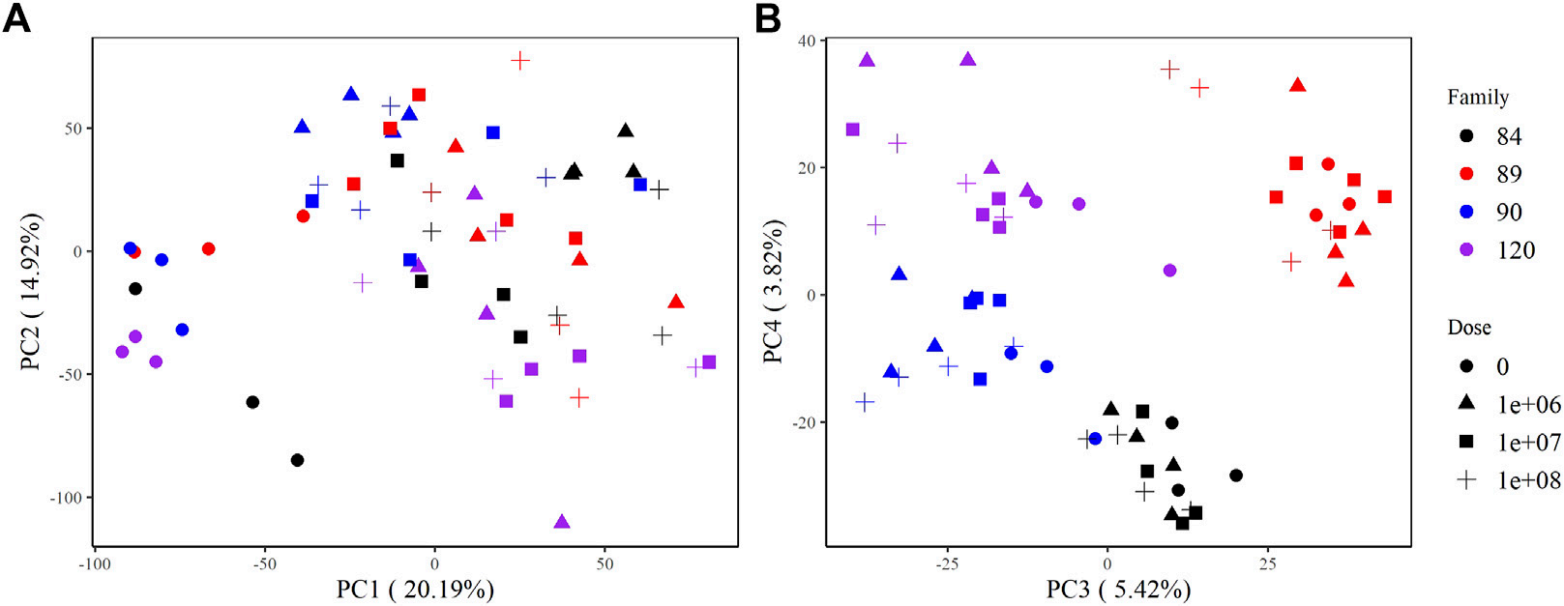
PCA plots of variance stabilizing transformed counts showing global gene expression patterns among samples. **(A)** PC1 and PC2 group samples by treatment (not dosed or dosed) and **(B)** PC3 and PC4 group samples by family.

The principal components analysis clearly discriminated samples by treatment based on overall expression along PC1, which explains >20% of the variance in the data. Zero-dose, field infected oysters formed a discrete cluster, while all other doses were indistinguishable from one another (Figure 2A). Samples also grouped by family along PC3 and PC4. Samples from sensitive families (90, 120) clustered together along PC3; however, we saw distinct separation between them along PC4. Samples from tolerant families (84, 89) formed unique clusters along both PC3 and PC4 (Figure 2B).

### 3.3 WGCNA

**FIGURE 3:**
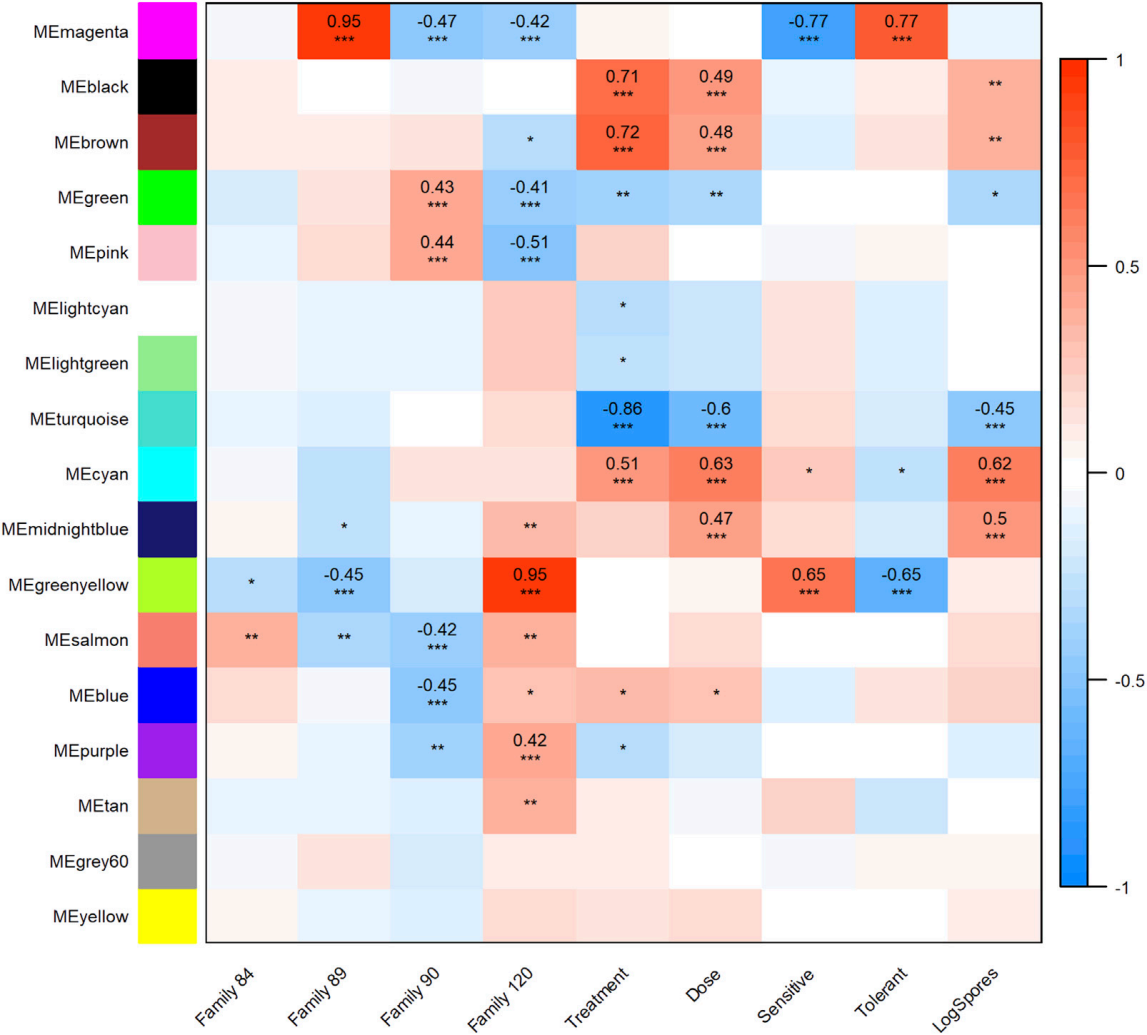
Weighted Gene Correlation Network Analysis module-trait relationship heat map. Trait (columns) to eigengene (row) correlation *p*-values ≤0.001 are represented as “***,” *p*-values ≤0.01 and >0.001 as “**,” and *p*-values ≤0.05 and >0.01 as “*”. The color scale shows the strength of each correlation, ranging from dark blue (−1) to dark red (1), correlation values are shown for module trait relationships that were highly significant.

All transcripts included in the WGCNA analysis were assigned to one of 17 modules (clusters of transcripts that were co-expressed across samples in our dataset). Twelve modules were highly and significantly correlated (*r* ^2^ ≥ 0.40 and *p*-value ≤0.001) with one or more traits of interest (Figure 3). Modules black, brown, and cyan were significantly positively correlated with treatment and dose; cyan was also positively correlated with (log) spores. Positive correlations between module midnightblue, dose and (log) spores were also detected. The turquoise module was negatively correlated with treatment, dose, and (log) spores. Modules magenta and greenyellow were both correlated with phenotype, but in opposite directions. These modules were also correlated with at least one family in each phenotype grouping. Given that the objectives of this study were to determine the effect of parasite dose on global gene expression and identify transcripts, gene networks, and gene ontologies that help define dermo tolerant phenotypes, we will discuss modules midnightblue, cyan, black, brown, turquoise, and magenta in greater detail below.

A broad and complex expression response to *P. marinus* treatment, dose, and infection intensity at day 7 post-injection was observed. Of the five modules significantly correlated with these traits, midnightblue best encapsulated the immune response to the parasite. It consisted of 287 transcripts, 190 and 170 of which had Blast2Go gene descriptions and GO annotations, respectively. “G-protein-coupled receptor signaling pathway,” “MyD88-dependent toll-like receptor signaling pathway,” “leukocyte activation,” “pattern recognition receptor signaling pathway,” and “defense response to bacterium” were among the significantly enriched biological process GO terms in this module (Supplementary Material S2). Transcripts with the highest module membership values (kME >0.8) included four tripartite motif-containing proteins (TRIM), two tumor necrosis factor ligand superfamily member 10-like proteins, hemicentin-2-like, E3 ubiquitin-protein ligase TRIM71-like, F-box only protein 30, GRB2-associated and regulator of MAPK protein-like, cis-aconitate decarboxylase, and transcriptional regulator Erg (Supplementary Material S3).

Cyan was another well-annotated module of interest with all but 41 of 335 transcripts described and 231 with assigned GO terms. Top transcripts in the cyan module (kME >0.80) were involved with the cell cycle, specifically the initiation and regulation of DNA replication (PCNA-associated factor, geminin isoform X2, DNA replication complex GINS protein P SF2-like, DNA polymerase alpha subunit B-like, and ribonucleoside-diphosphate reductase subunit M2) and chromosomal organization/division (various kinetochore proteins, vesicle-associated membrane protein 8-like, centromere protein O-like, polyamine-modulated factor 1-like, cyclin A, and transforming acidic coiled-coil-containing protein 3-like). Not surprisingly, the most enriched GO terms in this module also reflect important cell cycle and DNA replication-associated functions (Supplementary Material S2).

The brown (4,197 transcripts) and black (1,914 transcripts) modules were composed of transcripts involved in several diverse cellular, biosynthetic, and metabolic processes. For the brown module, 144 GO terms were enriched at a level *p* < 0.01. The most highly enriched (*p* < 0.001) biological processes included “cellular respiration,” “ATP metabolic process,” “peptide metabolic process,” “RNA metabolic process,” “RNA catabolic process,” “macromolecule biosynthetic process,” “RNA biosynthetic process,” “RNA processing,” “cellular response to stimulus,” and “cellular response to DNA damage stimulus” (Supplementary Material S2). Moreover, a large number (107) of ribosomal protein transcripts were among those with the highest kME values (Supplementary Material S3). Many of the top transcripts in the black module play a role in the formation and maintenance of cilia. For example, DPCD-like protein, intraflagellar transport protein 172 homolog, clusterin-associated protein 1, tubulin polyglutamylase complex subunit 1-like, B9 domain-containing protein 2-like, migration and invasion-inhibitory protein, and protein PIH1D3-like all had kME values >0.8. While the biological process GO term “cilium organization” was enriched in the black module, “nucleosome organization,” “DNA-templated transcription” and “protein-DNA complex subunit organization” were enriched at a higher significance level (Supplementary Material S2).

The largest module in the WGCNA analysis was turquoise with 17,835 transcripts. The strong negative correlation between the turquoise module and treatment, dose, and (log) spores implies a pattern of decreasing expression with increasing trait values, such that as dose and parasite load increase, expression of transcripts in the module decreases. Biological process GO terms for transport, protein modification, response to stimulus, and signaling were all enriched. Specifically, “intracellular signal transduction,” “regulation of Ras protein signal transduction,” “regulation of small GTPase mediated signal transduction,” “cell surface receptor signaling pathway,” and “WNT signaling pathway” were enriched at the highest significance level (Supplementary Material S2).

While the previous five modules summarized the general oyster transcriptomic response to *P. marinus*, the magenta module characterized the expression patterns of distinct families and phenotypes exposed to the parasite. Positively correlated with the tolerant phenotype and family 89 and negatively correlated with the sensitive phenotype, families 90 and 120, this module contained 1,619 transcripts, 483 of which had an “uncharacterized” Blast2GO description and 830 without a GO annotation. Described, annotated transcripts with the highest module membership (kME >0.8) included NADH dehydrogenase [ubiquinone] flavoprotein three mitochondrial, tripartite motif-containing protein (TRIM) 45-like, deleted in malignant brain tumors one protein-like, dynactin subunit 1-like, lysophospholipid acyltranserferase 7-like, beta-1,3-galactosyltransferase brn-like, oxidoreductase YajO, tripartite motif-containing protein 5-like, and tripartite motif-containing protein 2-like (Supplementary Material S3). Members of the TRIM protein family were well-represented in this module with 79 transcripts total. E3 ubiquitin ligase was another abundant family with 36 transcripts included in the magenta module. We also identified seven transcripts coding for serine protease inhibitors, 13 heat shock protein 70 (HSP70) 12A-like transcripts, and at least 21 transcripts potentially acting within the apoptosis pathway in oysters: toll-like receptors (TLR), tumor necrosis factor receptors (TNFR), baculoviral IAP repeat containing (BIRC) proteins, caspase 3, caspase 9, and putative inhibitors of apoptosis (Witkop et al., 2022a). Biological process GO terms “response to extracellular stimulus,” “response to external stimulus,” “pattern recognition receptor signaling pathway,” and “programmed cell death” were among those significantly functionally enriched in the magenta module (Supplementary Material S2).

### 3.4 Differential gene expression analysis with DESeq2

**FIGURE 4:**
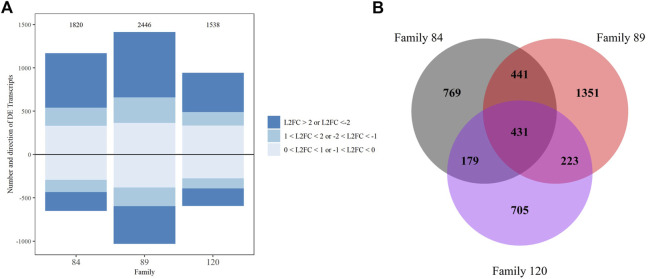
DESeq2 differential expression analysis results for family comparisons with family 90 as the reference, transcripts with false discovery rate corrected *p*-value <0.05 were considered significant. **(A)** Up and downregulated transcripts and their Log2 foldchange intensity, the total number is noted above each bar. **(B)** Venn diagram depicting overlap between comparisons.

Pairwise comparisons of gene expression between our most sensitive family (90) and each of the other families (84, 89, 120) dosed with 10^8^ *P. marinus* cells g^−1^ wet tissue weight detected a substantial number of DETs. More DETs were identified when comparing family 90 with tolerant families 84 and 89 (1,820 and 2,446, respectively) than with sensitive family 120 (1,538) which was expected given disparate phenotypes were contrasted (Figure 4A). The majority of DETs across all three comparisons had a L2FC > 1 or < −1. We also observed more upregulated than downregulated transcripts in each comparison. Among the most upregulated transcripts in family 84 relative to family 90 were several members of the complement C1q, TRIM, MEGF10, Von Willebrand factor D and EGF domain-containing protein, heat shock 70 kDa protein 12, and lectin families (Supplementary Material S4). The most enriched biological process GO terms associated with DETs from this comparison included “negative regulation of mitotic metaphase/anaphase transition,” “fatty acid alpha-oxidation,” “regulation of anion transport,” and “membrane assembly” (Supplementary Material S5). Many of the most highly upregulated transcripts in the comparison between families 89 and 90 were also members of complement C1q, TRIM, Von Willebrand factor D and EFC domain-containing protein, and heat shock 70 KDa protein 12. The MEGF10 family was well-represented among both up- and downregulated transcripts in this comparison (Supplementary Material S4). Biological process GO terms “programmed cell death” and “response to chemical” were enriched in the upregulated DET set while “glutamine biosynthetic and metabolic process” were overrepresented among downregulated DETs (Supplementary Material S5).

We also examined the number and composition of overlapping DETs across comparisons. Four hundred thirty-one DETs were shared by all three comparisons (84/90, 89/90 and 120/90). The fewest overlapping transcripts (179) were detected between the 84/90 and 120/90 DET lists. Not surprisingly, overlap was greatest (441) in the instance where differential expression was tested between either tolerant family (84 or 89) and family 90 (Figure 4B). These 441 transcripts are most likely to distinguish tolerant from sensitive phenotypes. Within this group, 102 transcripts were uncharacterized, three were differentially expressed in opposite directions, and 21 were differentially expressed in the same direction but had very disparate L2FC values (Supplementary Material S6). Included in the overlapping transcripts were 27 TRIM family members (16 with L2FC > 2 and 2 with L2FC < −2), 11 E3 ubiquitin protein ligases (3 each with L2FC > 2 and < −2), eight Von Willebrand factor D and EFC domain-containing proteins (7 with L2FC > 2), seven MEGF10 family members (6 with L2FC > 2 and 1 with L2FC < −2), and six complement C1q proteins (3 with L2FC > 2). Other highly expressed, shared, transcripts of interest included vezatin isoform X2, G-protein coupled receptors, cell death abnormality protein 1-like, and toll-like receptor 4. To visualize the potential functionality of the shared list of upregulated transcripts, we used OmicsBox’s combined graph option to make direct acyclic graphs for each GO category separately followed by the “sequences per GO term” function, to obtain the lowest child term (node) per branch. Biological Process GO terms “signal transduction,” “nucleic acid metabolic process,” and “ion transmembrane transport” were well-represented in the dataset (Table 2).

**TABLE 2:** Upregulated biological process child gene ontology terms for transcripts that were differentially expressed in families 84 and 89 DESeq2 comparisons.

GO term	GO id	Number of contributing sequences	Node score
Signal transduction	GO:0007165	10	14.67
Nucleic acid metabolic process	GO:0090304	10	14.59
Ion transmembrane transport	GO:0034220	7	9.96
Regulation of cellular metabolic process	GO:0031323	6	6.90
Cellular macromolecule metabolic process	GO:0044260	7	4.40
Cellular component assembly	GO:0022607	6	3.21
Phosphate-containing compound metabolic process	GO:0006796	6	3.07
Protein modification process	GO:0036211	6	2.57
Gene expression	GO:0010467	7	2.35
Regulation of macromolecule metabolic process	GO:0060255	7	1.85
Organic substance biosynthetic process	GO:1901576	8	1.32
Cellular nitrogen compound biosynthetic process	GO:0044271	6	0.83
Small molecule metabolic process	GO:0044281	6	0.74

**FIGURE 5:**
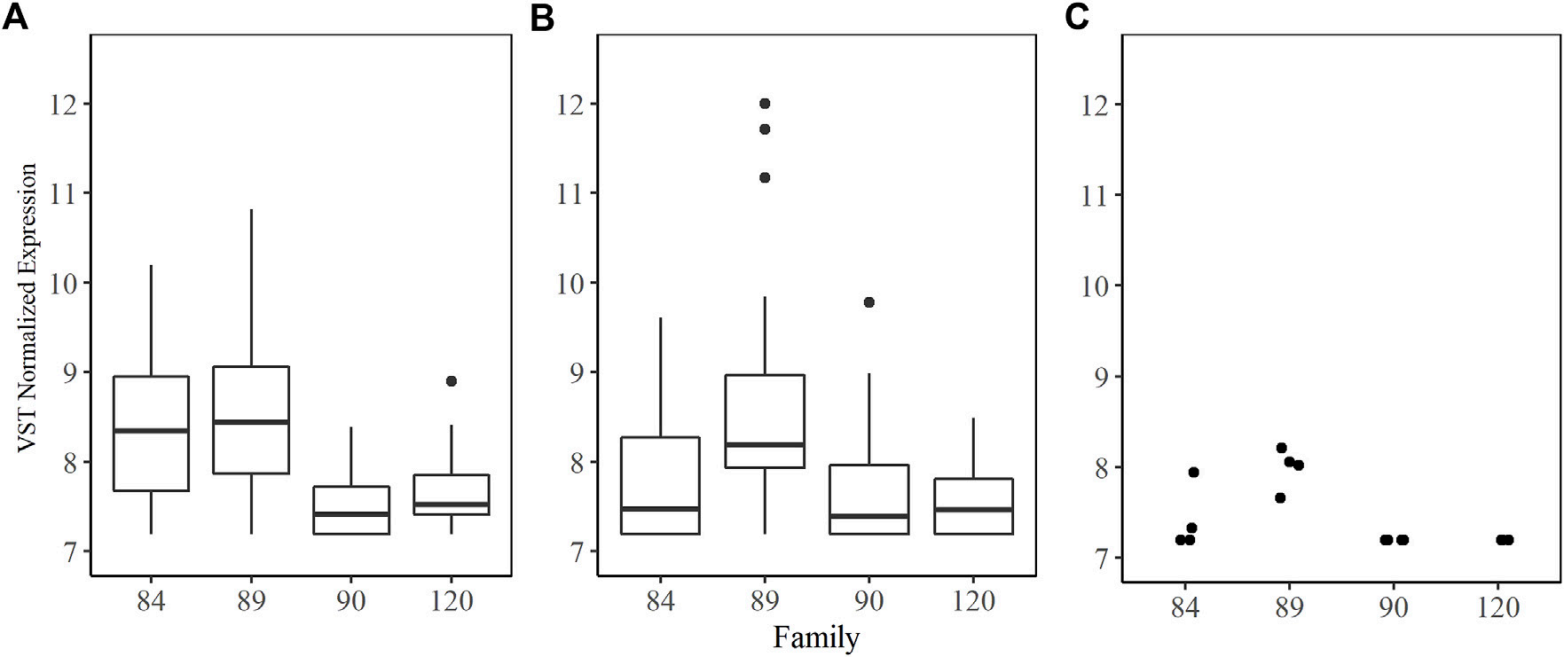
Variance stabilizing transformation normalized counts by family for **(A)** median expression of 11 tripartite motif-containing protein (TRIM) transcripts shared between magenta module and DESeq2 comparisons for family 84 and 89; **(B)** median expression of seven serine Protease Inhibitors (SPI) found in the magenta module; and **(C)** expression for serine protease inhibitor Cvsi-2 (XM_022434710.1) found in the magenta module.

Furthermore, there were 110 overlapping transcripts among the WGCNA magenta module member list and the DETs shared between the 84/90 and 89/90 comparisons. Over one-third of these were uncharacterized. Eleven TRIM proteins were identified, once again suggesting a role in the dermo tolerant phenotype. A box plot summarizing the VST-normalized expression values of these transcripts in each family confirms that they are consistently more highly expressed in the tolerant families (Figure 5A). Transcripts neurogenic notch homolog protein 1-like, stimulated by retinoic acid gene six protein-like, probable ATP-dependent helicase PF08-0048, ABC transporter F family member 4-like, vezantin, four E3 ubiquitin-protein ligases, and two MEGF10 were also magenta module members and had significantly higher expression (L2FC > 2) in both tolerant families compared to family 90.

Notably absent from this overlapping transcript list were serine protease inhibitors (SPI). A total of 79 different SPI transcripts were expressed in our entire dataset. They were distributed across nine WGCNA modules; two of which were positively correlated with treatment and dose (blue and brown), two of which were negatively correlated with treatment and dose (green and turquoise), and one positively correlated with the tolerant phenotype (magenta). The magenta module contained seven SPI transcripts (three kunitz-type serine protease inhibitors, two serine protease inhibitor Cvsi-2-like, one serine protease inhibitor dipetalogastin, and one kazal-type serine protease inhibitor), none of which were differentially expressed in the 84/90 comparison and only one (Cvsi-2 XM_022434710.1) was significantly differentially expressed in the 89/90 comparison at a low L2FC. There were three differentially expressed SPI between sensitive family 90 and tolerant family 89 and two differentially expressed between families 90 and 84, but in all cases the L2FC was less than 0.2. VST normalized expression of the seven SPIs in the magenta module did not differ significantly across families, although expression in family 89 was highly variable (Figure 5B). Serine protease inhibitor Cvsi-2 (XM_022434710.1) expression was similar among all family 89 samples and significantly higher than expression in families 90 and 120. Expression was varied within family 84 samples (Figure 5C).

## 4 Discussion

There is great potential for selective breeding to contribute to disease management in cultured and restored bivalve populations, but the target trait must be variable and well-defined. Several dermo response traits (field survival, parasite avoidance, and resistance) have already been characterized in a Mid-Atlantic eastern oyster breeding population. In this study, we focused on dermo tolerance because it is a stable trait that not only can improve industry productivity but also sustain free-standing oyster populations near aquaculture sites. By measuring multiple eastern oyster families’ fitness in a range of *P. marinus* concentrations, we directly assessed tolerance and demonstrated that genetic variation for the trait exists. These same families did not appear to vary in dermo resistance. To gain a better understanding of the relationship between parasite dose and host survival as well as the genetic mechanisms specifically underlying tolerance, we concentrated on four families, two sensitive and two tolerant, and evaluated global gene expression at increasing parasite loads in mantle tissue 7 days post-injection. Several significant correlations between dose and groups of transcripts with similar expression patterns among all samples sequenced were identified. Our results confirmed some of what others have reported with respect to the oyster innate immune response; however, they also provided novel, valuable, insight to aspects of oyster metabolism and cell proliferation that are influenced by infection intensity. A single cluster of transcripts with similar expression profiles (magenta module) was highly correlated with the tolerant phenotype. The transcripts and enriched GO categories contained within the cluster offer testable hypotheses for how tolerant oysters can coexist with *P. marinus*, primarily through preservation and effective utilization of apoptotic machinery. A separate differential gene expression analysis comparing each tolerant family to the most sensitive one produced a transcript set that overlapped considerably with members of the magenta module, underscoring the validity of our global gene expression analysis. We discuss our key findings in greater detail below.

Transcripts in module midnightblue appear to be involved in a coordinated immune response to *P. marinus* that is accentuated with increasing parasite load. Hemocytes, which are very abundant in mantle tissue, are the oyster’s primary immune cells and first line of defense against pathogens (Allam and Raftos, 2015). Therefore, it is no surprise that the GO term “leukocyte activation,” referring to heightened immune cell response, was significantly enriched in the midnightblue module. Hemocyte activation involves a variety of transmembrane receptors and signaling pathways, including the versatile G-protein coupled receptor and highly conserved Toll-like receptor pathways. “G-protein coupled receptor signaling” facilitates cellular responses to extracellular signals and regulates a diverse array of processes including cell migration, inflammation, and phagocytosis (Li et al., 2019; Gao et al., 2021). In addition to our study, upregulation of the G-protein coupled receptor signaling pathway has previously been observed in Pacific oyster spat exposed to the OsHV-1 virus (He et al., 2015; Guo and Ford, 2016) and 1 year old eastern oyster seed infected with *P. marinus* (Proestou and Sullivan, 2020). Moreover, the G-protein coupled receptor 157-like transcript, which had the second highest module membership in our midnightblue WGCNA module was found to be significantly upregulated in hemolymph from adult *O. edulis* oysters lightly infected with the parasite *B. exitosa*, highlighting its potential importance in the dermo response (Martín-Gómez et al., 2012).

Toll-like receptors (TLR) are a family of pattern recognition receptors that are significantly expanded in bivalve genomes (Zhang et al., 2012; Toubiana et al., 2013; Zhang L.et al., 2014; Xing et al., 2017). Pathogen recognition stimulates signaling along MyD88-dependent pathways which culminate in the production of antimicrobial peptides, lysozymes, and other immune effector molecules (Toubiana et al., 2014; Gerdol et al., 2018). Several studies have reported overexpression of the TLR pathway in disease-challenged bivalves. Toll-like receptors 4, 5, and 6, as well as interleukin 17-like, were upregulated in *Mytilus galloprovincialis* gill tissue after bath exposure to the pathogenic bacteria *V. splendidus* (Saco et al., 2020). Upregulation of the MyD88-dependent signaling pathway was also observed in Venus clam hemocytes exposed to gram-positive (*Micrococcus luteus*) and gram-negative (*V. anguillarum*) bacteria and knockdown of the toll-like receptor 13 gene blocked transcription of downstream pathway members (Ren et al., 2017). Immune priming of juvenile Pacific oysters with the viral mimic, poly (I-C), resulted in sustained elevated expression of TLR pathway members and conferred full protection against OsHV-1 (Lafont et al., 2020). Thus, our results contribute additional evidence that MyD88-dependent TLR signaling pathways play a critical role in the general bivalve immune response to a variety of pathogens.

Tumor necrosis factor (TNF) signaling was also enriched in the midnightblue module, with a receptor and three TNF ligand superfamily member 10-like (also referred to as TRAIL) transcripts among those with the highest module membership. TNF receptors and their ligands have been described in all eukaryotes. Despite the incredible diversity in number of TNF-related genes and low sequence similarity between and within taxonomic groups, their functionality appears to be conserved (Quistad et al., 2014; Quistad and Traylor-Knowles, 2016). Some TNF receptors contain intracellular death domains (DD) whose presence dictates ligand binding. In humans, TRAIL binds to death domains that can either induce caspase-dependent programmed cell death (apoptosis) in transformed/tumor cells or activate Nuclear Factor kappa B (NFκB) signaling which mediates the cellular inflammatory response (Sonar and Lal, 2015). Recent studies suggest similar functionality in molluscs. A gene coding for a 29.5 kDa TRAIL (TgTNFSF10) from the blood clam (*Telillarca granosa*) was cloned, characterized, and shown to be significantly upregulated in immune-stimulated hemocytes. Moreover, apoptotic rates in clam hemocytes and human hepatoma (HEpG2) cells treated with recombinant TgTNFSF10 were significantly higher (55.16% and 42.3% respectively) than in untreated cells, confirming this gene’s capacity to induce cell death (Liu et al., 2021). In European flat oysters, the intensity of field-acquired *Bonamia* infections was correlated with TNF ligand superfamily-like transcript levels (Martín-Gómez et al., 2012). Differential expression of several TNF ligands and receptors was also observed in adult Pacific oysters challenged with gram negative bacteria, although the level and direction of expression differences varied over time (Zhang et al., 2015). The enriched signaling pathways in the midnightblue module play multiple, diverse roles in the oyster innate immune response, but all can initiate apoptosis. In addition to maintaining immune homeostasis, apoptosis can provide protection against pathogens when infected hemocytes are eliminated in the absence of inflammation (Sokolova, 2009).

To maintain the increased immune activity stimulated by pathogens, hemocytes must proliferate. Our results indicate this occurred in our treated oysters since the vast majority of significantly enriched biological process GO terms and transcripts with the highest membership in the cyan module were involved in the cell cycle (S and M phases). As with the midnightblue module, expression of transcripts in the cyan module increased with parasite load. Heightened cell cycle activity in response to infection has been observed in other oyster species as well. For example, two-year-old Pacific oysters treated with heat-killed bacteria produced double the number of hemocytes than control oysters within a 12-h period. The same level of hemocyte production and increased phagocytic activity were observed in half the time when oysters were secondarily exposed to live bacteria (Zhang T. et al., 2014). A closer look at the mechanisms of hematopoiesis within the context of immune priming in the Pacific oyster demonstrated dramatically elevated numbers of dividing cells and DNA synthesis in gill tissue post-challenge, suggesting pallial tissues may be the site of hematopoiesis (Li et al., 2017). Contents of the black module also support enhanced hemocyte proliferation in oysters treated with *P. marinus* regardless of parasite load. Enriched GO terms “nucleosome organization,” “microtubule organizing center process,” and “DNA-templated transcription” reflect cell cycle processes that occur during the G phase (Deniz et al., 2016).

It follows that stimulation of the innate immune response and immune cell proliferation would promote a rise in some metabolic activity. The most significantly enriched GO terms in the brown module, which was positively correlated with treatment and dose (but not (log) spores), were macromolecule biosynthetic processes (specifically RNA and protein synthesis). An uptick in protein synthesis and the resulting elevation of energy expenditure are common responses to stressors (Sokolova, 2021). Metabolic processes are not only necessary for growth and reproductive homeostasis, but also required to synthesize a large population of proteins critical for protecting cells and tissues from injury and mounting effective immune defenses (Wang et al., 2019). A study looking at both eastern and Pacific oyster (*C. gigas*) responses to *P. marinus* challenge, (Tanguy et al., 2004), corroborated what we saw in the brown module: patterns of differential expression suggesting increased energetic demands due to parasitic infection. Physiological condition and the ability to meet additional metabolic needs was also positively correlated with survival in Pacific oysters exposed to OsHV-1 (Fuhrmann et al., 2018).

While signaling and metabolic activity promoting an immune response increased with dose and parasite load, many other normal cellular activities (summarized by the enriched GO terms in the turquoise module) decreased. For example, we observed downregulation of GO terms associated with the Ras superfamily of small GTPases which control cell growth, cytoskeleton integrity, cell-cell adhesion, and cellular differentiation (Goitre et al., 2014). Similarly, as dose and (log) spores increased, expression of transcripts involved in Wnt signaling, which mediates proper formation and maintenance of important tissues by controlling cytoskeleton integrity, cell proliferation and migration, and cell fate determination, decreased (Teo and Kahn, 2010). GO terms involved with cytoskeleton organization were also enriched among downregulated transcripts in a sensitive eastern oyster family after experimental exposure to *P. marinus* (Proestou and Sullivan, 2020). Thus, our observations of reduced cellular processes in more infected oysters were in line with the characteristic tissue wasting that diseased oysters experience and may represent the delicate energetic balance between regulating normal cellular activity and heightened immune function.

As mentioned above, apoptosis is a keystone of cellular innate immune processes in bivalves and has been identified specifically as a mechanism by which *Perkinsus* species interact with their hosts. Previous studies examining apoptosis in eastern oyster hemocytes treated with virulent *P. marinus* strains reported significant and sustained decreases in cell death from basal levels within hours of exposure (Hughes et al., 2010; Witkop et al., 2022b). Increased parasite phagocytosis by hemocytes coupled with reduced cell death and high infection intensities suggests *P. marinus* interferes with apoptotic machinery to establish itself in host tissues (Hughes et al., 2010). The exact interference mechanism is unknown, but it appears to be caspase-independent and may involve parasite stimulation of host inhibitor of apoptosis gene expression (Witkop et al., 2022b). *P. marinus* also was shown to overexpress antioxidants (superoxide dismutase and peroxiredoxin) known to suppress apoptosis in the presence of oyster hemocytes (Lau et al., 2018). Although the evidence supporting *P. marinus* suppression of apoptosis is strong, other studies indicate bivalves can successfully utilize apoptosis pathways to resist or contain *Perkinsus* diseases. In both eastern oyster and manila clam, apoptosis related genes were overexpressed in response to low-level (<(log) spores 4) infections (Romero et al., 2015; Sullivan and Proestou, 2021).

By characterizing disparate dermo response phenotypes and comparing their gene expression patterns, we gained additional insight to the role apoptosis plays in this host-parasite interaction. The GO term “programmed cell death” was enriched in WGCNA modules magenta (which was positively correlated with the tolerant phenotype and family 89) and blue (which was negatively correlated with sensitive family 90) as well as DETs upregulated in family 89 compared to family 90. Consistent signal from both global gene expression analysis methods highlights the importance of apoptosis pathway execution in discriminating tolerant from sensitive phenotypes. But how do tolerant eastern oysters circumvent *P. marinus* suppression of apoptosis? In addition to antioxidants, *P. marinus* secrete serine proteases that were shown to inhibit eastern oyster hemocyte migration, lysozyme activity, and haemagglutination in *vitro* experiments and increase *in vivo* infections (Garreis et al., 1996; La Peyre et al., 1996). In other systems, serine proteases can modulate apoptosis independent of caspases (Egger et al., 2003), but this has not been investigated in oysters. Host serine protease inhibitors that neutralize *Perkinsus* virulence factors and decrease infection intensity have been identified (La Peyre et al., 2010; Nikapitiya et al., 2014; Xue, 2019). Protease inhibitor activity was significantly enriched in upregulated transcripts of a resistant oyster family injected with the parasite (Proestou and Sullivan, 2020). The hypothesis that protease inhibitors protect the host is strongly supported in the literature; however, the specific mode of action and role in mediating apoptosis warrant further study. In our dataset, elevated expression of serine protease inhibitor Cvsi-2 was consistent in tolerant family 89 but quite variable in tolerant family 84, suggesting that additional effector molecules are involved in dermo tolerance.

TRIM proteins are a large, conserved family of RING-domain containing E3 ubiquitin ligases that mediate post-translational modification *via* ubiquitination (Kawai and Akira, 2011). Because most proteins in the cell will be marked for modification/degradation, TRIMs are expressed at high levels and affect nearly all cellular processes including the inflammatory response, cell cycle progression, and apoptosis (Gushchina et al., 2018). TRIMs modulate the immune response by interacting with key signaling molecules (e.g., TLR) and tagging pathogen proteins for degradation (Patil and Li, 2019). In mammalian systems, some TRIMs interact with interferons and inhibit viruses by disrupting their life cycle (Ozato et al., 2008). Our data suggest that differential expression of TRIMs plays a role in conferring Dermo tolerance in the eastern oyster. TRIMs were well-represented among transcripts with high magenta module membership and detected DETs between tolerant and sensitive phenotypes. One-10th of the characterized transcripts shared between the magenta WGCNA module and DETs identified in both 84/90 and 89/90 DESeq2 comparisons were TRIMs; primarily TRIM two and 3-like. Two TRIM proteins involved in anti-viral (CgTRIM1) and anti-bacterial (ChTRIM9) responses recently were characterized in *C. gigas* and *C. hongkongensis* (Liu et al., 2016; Wang et al., 2021). TRIMs were upregulated in OsHV-1 challenged and POMS-resistant Pacific oysters compared to control and sensitive individuals (He et al., 2015; de Lorgeril et al., 2020). The most abundant immune-related transcripts identified in an experiment comparing *Bonamia* sensitive and resistant European flat oysters were also TRIMs (Pardo et al., 2016). While it is beyond the scope of this work to speculate on specific TRIM protein modes of action in bivalves, their function in the oyster immune response and alterations in sequence and expression patterns underlying dermo-tolerant phenotypes should be investigated further.

## 5 Conclusion

Tolerance, the ability of host organisms to limit negative impacts on fitness by a pathogen, has important implications for disease management in wild and cultured shellfish populations. Until now, this critical disease response trait has been poorly defined and/or conflated with other mechanisms, namely resistance. Our study represents the first attempt to clearly define and measure disease tolerance in a commercially important bivalve mollusc. Variation in the rate of fitness decline with increasing parasite load was observed among families tested, suggesting dermo tolerance can be targeted for selection. We saw little evidence for variation in dermo resistance in the same families. The methods used to evaluate tolerance (simultaneous laboratory challenge of several distinct genetic groups with multiple doses of the parasite) may not be practical for routine, family-based, selective breeding operations. Therefore, we performed global transcriptome analyses not only to characterize the eastern oyster genomic response to increasing *P. marinus* dose/parasite load but also to identify specific differences in gene expression between tolerant and sensitive oysters that could aid the selection process. As expected, oyster hemocyte activation *via* various cellular signaling pathways, cell proliferation, and immune-related RNA and protein synthesis increased with increasing parasite load while transcripts associated with normal cellular activity (growth, integrity, differentiation) decreased. Previous studies describing eastern oyster, *P. marinus* interactions present a scenario where oyster hemocytes migrate toward the site of infection and phagocytose parasitic cells. Once inside hemocytes, parasites suppress controlled cell death and proliferate ultimately causing them to burst, thereby facilitating infection spread. Our results indicate that the apoptotic pathway is fully executed in tolerant, but not sensitive oysters exposed to *P. marinus*. The mechanism by which tolerant eastern oysters overcome *P. marinus* suppression of apoptosis is unclear, but may involve serine protease inhibitors, tripartite motif-containing proteins, or both. Furthermore, our pairwise comparisons of gene expression profiles from two different tolerant families with that of a highly sensitive family demonstrated stronger serine protease inhibition and apoptotic activity signals in one of the tolerant families than the other, suggesting there are multiple avenues by which dermo tolerance can be achieved. Exploring mode of action through functional assays as well examining polymorphisms in these genes will be the focus of future work in order to develop a simpler way to breed for dermo tolerance.

## Data Availability

The data presented in the study are deposited in the NCBI short read archive repository under accession # PRJNA894694.
