# Supplementary material for: Understanding Crassostrea virginica tolerance of Perkinsus marinus through global gene expression analysis
Source: Front Genet. 2023 Jan 19;14:1054558. doi: 10.3389/fgene.2023.1054558 (PMC9892467; doi:10.3389/fgene.2023.1054558)

Supplemental 5

Hierarchical clustering trees created with standard mode for adaptive clustering combined with a Mann-Whitney U (MWU) test of DESeq2 results.


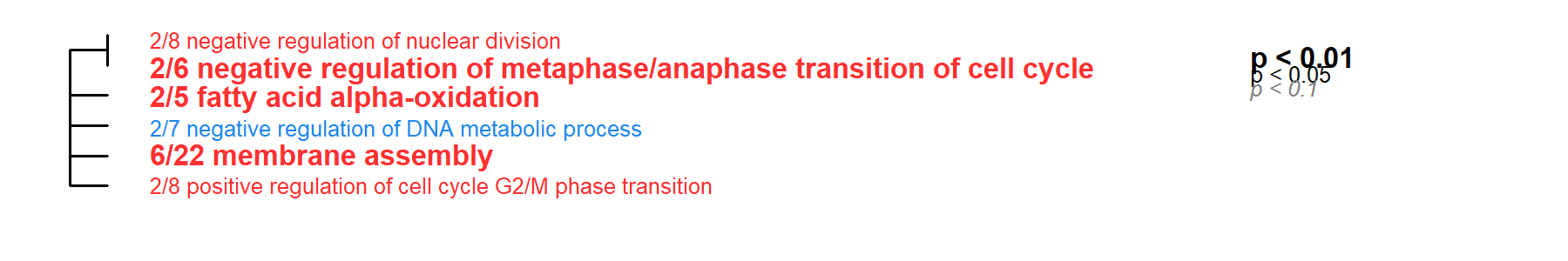
MWU 84/90 Biological Process

MWU 84/90 Molecular Function


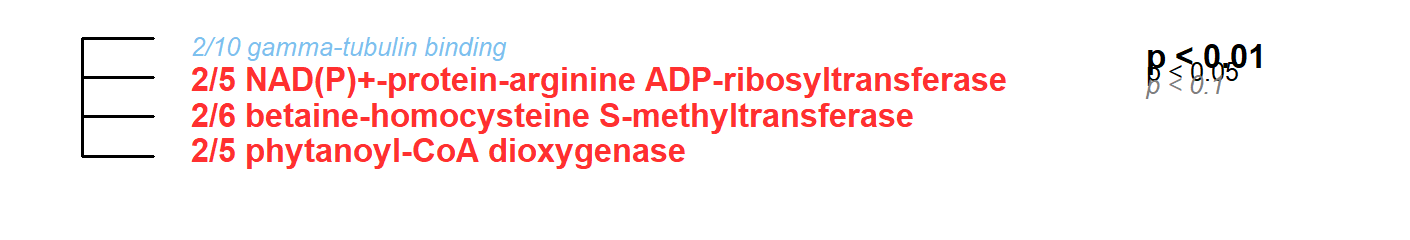


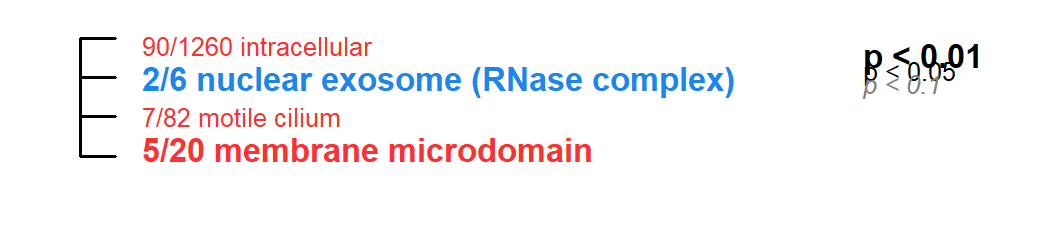
MWU 84/90 Cellular Component

MWU 89/90 Biological Process


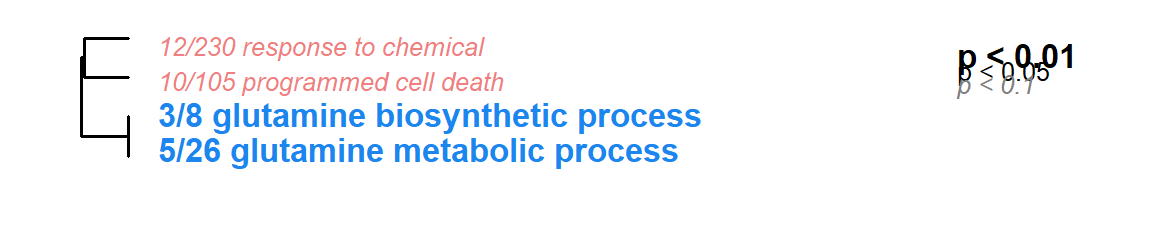


MWU 89/90 Molecular Function


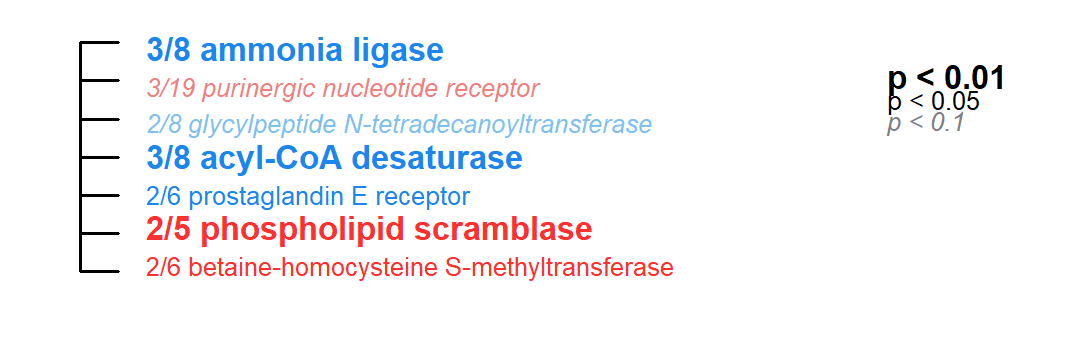


MWU 89/90 Cellular Component


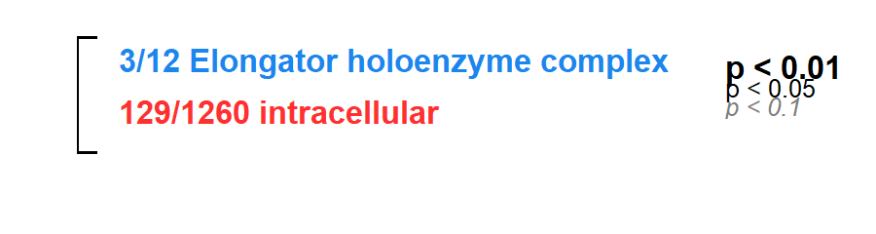


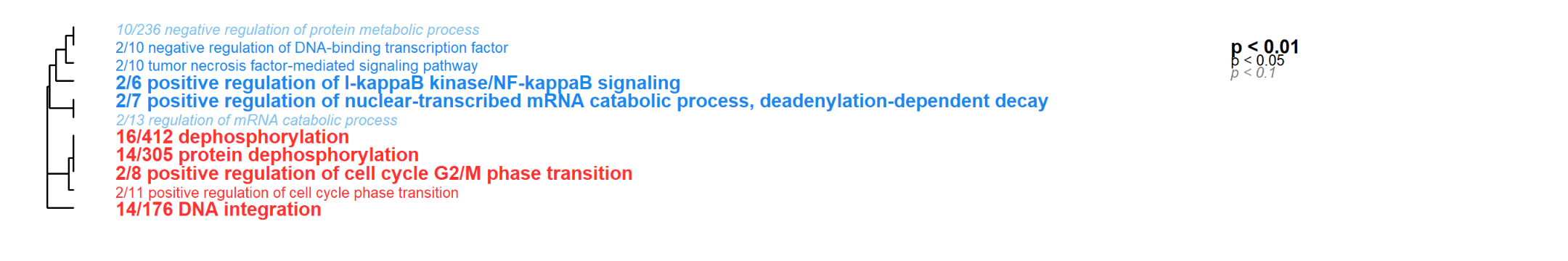
MWU 120/90 Biological Process

MWU 120/90 Molecular Function


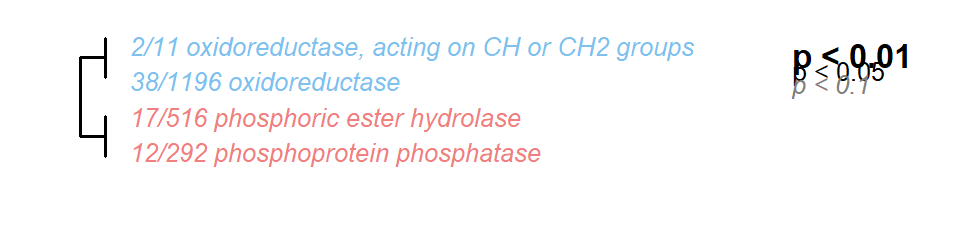

Supplement: Supplementary file 3 [file DataSheet5.docx]
