# Supplementary material for: Understanding Crassostrea virginica tolerance of Perkinsus marinus through global gene expression analysis
Source: Front Genet. 2023 Jan 19;14:1054558. doi: 10.3389/fgene.2023.1054558 (PMC9892467; doi:10.3389/fgene.2023.1054558)
Supplement: Supplementary file 5 [file DataSheet1.PDF]

```
#Supplemental 1, example code for:
#	1) PCA
#	2) WGCNA
#	3) MWU test for WGCNA magenta module biological process
#	4) DESeq2 for differential expression analysis with family 90 as reference
#	5) MWU test for differential expression analysis results
#	6) Venn Diagram

#1) PCA ####

#The following is a list of libraries used.

library(tidyverse)
library(dplyr)
library(plyr)
library(readtext)
library("DESeq2")
library(apeglm)
library(ggplot2)

#The following code was used to create PCA plots using DESeq2' variance stabilizing transformation (vst), the function "prcomp" for PCA, and ggplot2 to make plots
#Please refer to the Bioconductor guide "Analyzing RNA-seq data with DESeq2" for more details on the DESeq2 protocol
#(https://bioconductor.org/packages/release/bioc/vignettes/DESeq2/inst/doc/DESeq2.html)

#DESeq2 dataset is needed to run vst
#cts = count data for all samples (from CLC Workbench "RNASeq analysis"; please see text for details) in matrix format, each row = transcript and each column = sample
#coldata = metadata for all samples

#For VST blind = FALSE, we need to make a column of categories for the DESeq dataset design that will be used for normalizing.
coldata$Family_Dose <- as.factor(paste(coldata$Fam, coldata$Dose, sep="_"))

#make DESeq dataset
dds <- DESeqDataSetFromMatrix(countData = cts,
                              colData = coldata,
                              design = ~ Family_Dose)
dds
```

```
#transform dataset
vsd <- vst(dds, blind=FALSE)

#format results for prcomp
vsd_mat <- assay(vsd)

#run prcomp
pca <- prcomp(t(vsd_mat))

#bind results with metadata table
df <- cbind(coldata, pca$x)

#calculate percentage of variance explained for labeling
percentage <- round(pca$sdev^2 / sum(pca$sdev^2) * 100, 2)
percentage <- paste(colnames(pca$x), "(", paste( as.character(percentage), "%", ")", sep="") )

#make plots
PCA <- ggplot(df) + geom_point(aes(x=PC1, y=PC2, shape = as.factor(Dose), color=as.factor(Fam)), size=2.5)+theme_classic()+
  scale_color_manual(values = c("black","red", "blue", "purple"))+
  theme( legend.position = "none",  plot.title=element_text(size=15, family="serif")) +
  guides(shape=guide_legend(title="Dose", title.position = "top"),color=guide_legend(title="Family", title.position = "top"))+scale_size(10)+
  xlab(percentage[1]) + ylab(percentage[2])+theme(panel.background=element_blank(),panel.grid=element_blank(),panel.border=element_rect(fill=NA),
        text=element_text(family="serif",size=10),
        axis.title.y=element_text(family="serif",size=12),axis.title.x=element_text(family="serif",size=12))+ggtitle("A")

PCB <- ggplot(df) + geom_point(aes(x=PC3, y=PC4, shape = as.factor(Dose), color=as.factor(Fam)), size = 2.5)+theme_classic()+
  scale_color_manual(values = c("black","red", "blue", "purple"))+
  theme( legend.position = "right", legend.text=element_text(size=12, family="serif"),plot.title=element_text(size=15, family="serif")) +
  guides(shape=guide_legend(title="Dose", title.position = "top"),color=guide_legend(title="Family", title.position = "top"))+
  xlab(percentage[3]) + ylab(percentage[4])+theme(panel.background=element_blank(),panel.grid=element_blank(),panel.border=element_rect(fill=NA),
        text=element_text(family="serif",size=10),
        axis.title.y=element_text(family="serif",size=12),axis.title.x=element_text(family="serif",size=12))+ggtitle("B")

png("figures/PCA_multiplot_PC1PC2_PC3PC4_masked_publication_Dose_Fam2.png", width = 10, height = 4, units = 'in', res = 300)
grid.arrange(PCA,PCB,ncol=2, widths=8:9)
dev.off()
```

```
################################################################
#2) WGCNA ####
#The following script was used for weighted gene correlation network analysis (WGCNA), To view instructions and FAQ for WGCNA please visit:
#https://horvath.genetics.ucla.edu/html/CoexpressionNetwork/Rpackages/WGCNA/index.html

#The following is a list of libraries used.
library(WGCNA)
library(tidyverse)
library(plyr)
library(dplyr)
library(readtext)
library("DESeq2")
library(flashClust)

#cts = count data for all samples (from CLC Workbench "RNASeq analysis"; please see text for details) in matrix format, each row = transcript and each column = sample
#coldata = metadata for all samples

#this corrects an error associated with multiple packages using "cor"
cor <- WGCNA::cor

# The following setting is important, do not omit.
options(stringsAsFactors = FALSE)

#For WGCNA format coldata to be only numeric

coldata$Fam84 <- ifelse(coldata$Fam == "84", 1, 0)

coldata$Fam89 <- ifelse(coldata$Fam == "89", 1, 0)

coldata$Fam90 <- ifelse(coldata$Fam == "90", 1, 0)

coldata$Fam120 <- ifelse(coldata$Fam == "120", 1, 0)

coldata$Treatment <- ifelse(coldata$Treat == "INJ", 1, 0)

coldata$Dose2 <- ifelse(coldata$Dose == "1e+06", 1,
                        ifelse(coldata$Dose == "1e+07", 2,
```

```r
                              ifelse(coldata$Dose == "1e+08", 3, 0)))

coldata$Sensitive <- ifelse(coldata$Fam == "90" | coldata$Fam == "120", 1, 0)

coldata$Tolerant <- ifelse(coldata$Fam == "84" |  coldata$Fam == "89", 1, 0)

#For VST blind = FALSE, we need to make a column of categories for the DESeq dataset design that will be used for normalizing.
coldata$Family_Dose <- as.factor(paste(coldata$Fam, coldata$Dose, sep="_"))


#removing low counts, which is recommended.
#to get list of transcripts that passed low threshold count (10 or more) for any individual family

FamList <-  c("84", "89", "90", "120")

for (fam in FamList)
{
  meta <- coldata %>% subset(coldata$Fam==fam)
  count <- cts[, rownames(meta)]
  keep <- rowSums(count) >= 10
  newcount <- count[keep,] %>% rownames_to_column()
  assign(paste0("final",fam), newcount)
}

A <- merge(final84, final89, by="rowname", all=TRUE)
B <- merge(A, final90, by="rowname", all=TRUE)
exp <- merge(B, final120, by="rowname", all=TRUE)%>% select(rowname)

#to remove low counts keep only transcripts that pass the threshold
cts <- cts %>% rownames_to_column()  #changing the rownames to a column so we can merge
cts <- merge(exp, cts, by="XM")#merging so that we only keep XM IDs that were in the list of "highly" expressed transcripts
cts <- column_to_rownames(cts, var="rowname")#putting the rownames back

#the two lines of code below return "true" if column names and row names match for our metadata and count data
#(makes sure we have metadata for every sample in the count matrix and vis versa)
all(rownames(coldata) %in% colnames(cts))
all(rownames(coldata)==colnames(cts))
```

```
#select columns made above and columns to include in the analysis / removing unwanted columns.
coldata <- coldata %>%
  dplyr::select("Fam84", "Fam89", "Fam90",  "Fam120", "Treatment", "Dose2", "Sensitive", "Tolerant", "LogSpores", "Family_Dose")

#building the DEseq2 design to normalize with vst function and reduce over correcting data - https://support.bioconductor.org/p/115583/
dds <- DESeqDataSetFromMatrix(countData = cts,
                              colData = coldata,
                              design = ~ Family_Dose)
dds

#run vst = variance stabilizing transformation
vsd <- vst(dds, blind=FALSE) #transform the data

#get the transformed counts back into a matrix format
vsd_mat <- assay(vsd)

#remove "Family_Dose" from column data
coldata <- coldata %>%
  dplyr::select("Fam84", "Fam89", "Fam90",  "Fam120", "Treatment", "Dose2", "Sensitive", "Tolerant", "LogSpores")

#Need to format my matrix to fit WGCNA requirements, use the "t" function to invert my matrix
Cdata <- as.data.frame(t(vsd_mat))
#We first check for genes and samples with too many missing values:
gsg = goodSamplesGenes(Cdata, verbose = 3);
gsg$allOK
#TRUE
#If the last statement returns TRUE, all genes have passed the cuts.

#Next cluster the samples (in contrast to clustering genes that will come later) to see if there are any obvious outliers

# sample network based on squared Euclidean distance, note that the data is transposed
A=adjacency(t(Cdata),type="distance")

# this calculates the whole network connectivity
k=as.numeric(apply(A,2,sum))-1
```

```
# standardized connectivity
Z.k=scale(k)

# Designate samples as outlying if their Z.k value is below the threshold
thresholdZ.k=-3

# the color vector indicates outlyingness (red)
outlierColor=ifelse(Z.k<thresholdZ.k,"red","black")

# calculate the cluster tree using flashClust or hclust
sampleTree = flashClust(as.dist(1-A), method = "average")

# Convert traits to a color representation: where red indicates high values
traitColors=data.frame(numbers2colors(coldata,signed=FALSE))
dimnames(traitColors)[[2]]=paste(names(coldata),"C",sep="")
datColors=data.frame(outlierC=outlierColor,traitColors)

# Plot the sample dendrogram and the colors underneath.
plotDendroAndColors(sampleTree,groupLabels=names(datColors),
        colors=datColors,main="Sample dendrogram and trait heatmap")

#No outliers were found

# Allow multi-threading within WGCNA.
# Caution: skip this line if you run RStudio or other third-party R environments.
enableWGCNAThreads()

## Choose a set of soft-thresholding powers####
powers = c(c(1:10), seq(from = 12, to=20, by=2)) #from 1 to 20
# Call the network topology analysis function
sft = pickSoftThreshold(Cdata, powerVector = powers, verbose = 5, networkType = "signed")

#choose softthreshold power of 8

#calculate the weighted adjacency matrix, using the power 8:
A = adjacency(Cdata, power = 8, type="signed")
```

```
#calculate dissimilarity based on the topological overlap matrix
dissTOM =TOMdist(A, TOMType = "signed")

#hierarchical clustering
geneTree = flashClust(as.dist(dissTOM),method="average")

# Plot the resulting clustering tree (dendrogram)
sizeGrWindow(12,9)
plot(geneTree, xlab="", sub="", main = "Gene clustering on TOM-based dissimilarity", labels = FALSE, hang = 0.04);

# here we define the modules by cutting branches
dynamicMods_ds2=cutreeDynamic(dendro=geneTree,distM=dissTOM, method="hybrid", deepSplit=2, pamRespectsDendro=FALSE, minClusterSize=30)
table(dynamicMods_ds2)

# Convert numeric labels into colors
dynamicColors = labels2colors(dynamicMods_ds2)
table(dynamicColors)

# Plot the dendrogram and colors underneath
sizeGrWindow(8,6)
plotDendroAndColors(geneTree, dynamicColors, "Dynamic Tree Cut",
        dendroLabels = FALSE, hang = 0.03,
        addGuide = TRUE, guideHang = 0.05,
         main = "Gene dendrogram and module colors")

# Calculate eigengenes
MEList = moduleEigengenes(Cdata, colors = dynamicColors )
MEs = MEList$eigengenes

# Calculate dissimilarity of module eigengenes
MEDiss = 1-cor(MEs);

# Cluster module eigengenes
METree = hclust(as.dist(MEDiss), method = "average");

# Plot the result
sizeGrWindow(7, 6)
```

```
plot(METree, main = "Clustering of module eigengenes", xlab = "", sub = "")

# calculate the module merging threshold using the number of samples (in this case, 61)
dynamicMergeCut(61)
#0.1777744

#set threshold
MEDissThres =  0.1777744

# Plot the cut line into the dendrogram
abline(h=MEDissThres, col = "red")

# Call an automatic merging function, dynamicColors from above calculation
merge = mergeCloseModules(Cdata, dynamicColors, cutHeight = MEDissThres, verbose = 3)

# The merged module colors
mergedColors = merge$colors;
# Eigengenes of the new merged modules:
mergedMEs = merge$newMEs;

table(mergedColors)
table3 = table(mergedColors)
write.csv(table3 , file="data_out/Final_module_table.csv")

#To see what the merging did to the module colors, plot the gene dendrogram again, with the original and merged module colors underneath
sizeGrWindow(12, 9)
plotDendroAndColors(geneTree, cbind(dynamicColors, mergedColors),
        c("Dynamic Tree Cut", "Merged dynamic"),
        dendroLabels = FALSE, hang = 0.03,
        addGuide = TRUE, guideHang = 0.05)

# Rename to moduleColors
moduleColors = mergedColors
# Construct numerical labels corresponding to the colors
colorOrder = c("grey", standardColors(50));
moduleLabels = match(moduleColors, colorOrder)-1;
MEs = mergedMEs;
```

```
#save work
save(MEs, moduleLabels, moduleColors, geneTree, file = "data_out/AllData_Hovarth_Oyster_02-StepWiseNetwork_Signed_power8_Pearson_DS2.RData")

# Define numbers of genes and samples
nGenes = ncol(Cdata);
nSamples = nrow(Cdata);
# Recalculate MEs with color labels
MEs0 = moduleEigengenes(Cdata, moduleColors)$eigengenes
MEs = orderMEs(MEs0)
moduleTraitCor = cor(MEs, coldata, use = "p");
moduleTraitPvalue = corPvalueStudent(moduleTraitCor, nSamples);

##Calculate module membership using KME and Gene Significance for traits

#calculate the module membership values (aka module eigengene based connectivity kME):
datKME=signedKME(Cdata, MEs)

#Gene Significance
datGS.Traits=data.frame(cor(Cdata,coldata,use="p"))

#dataframe with both KME and GS info
# set names in the dataset as Transcript
Transcript = names(Cdata)
TranscriptInfo <- data.frame(XM=Transcript,
                             moduleColor = moduleColors,
                             datKME,
                             datGS.Traits)

#add annotation data to TranscriptInfo, see text for B2G information on how annotation was created (ann_XP_XM)
TranscriptInfo_ann <- merge(TranscriptInfo, ann_XP_XM, by = "XM")

##export module transcript lists with KME, GS, and annotation information
table(moduleColors)
Modules = c("black","blue","brown","cyan","green","greenyellow","grey60","lightcyan",
            "lightgreen","magenta", "midnightblue", "pink","purple", "salmon", "tan", turquoise", "yellow")
```

```
for (module in Modules)
{
  # Select module Transcripts
  modTranscript <-  (moduleColors==module)
  # Get their transcript IDs
  IDs <-  Transcript[modTranscript]%>% tbl_df();
  names(IDs)[names(IDs)=="value"] <- "XM";
  IDs_ann <-  merge(IDs,  TranscriptInfo_ann, by="XM" );
  IDs_ann <-  IDs_ann %>% dplyr::select("XM", "XP","moduleColor", paste("kME",module, sep=""),"Fam84", "Fam89", "Fam90",  "Fam120", "Treatment", "Dose2",
                                        "Sensitive", "Tolerant", "LogSpores" "Blast2Go_description", "Annotation.GO.ID", "Annotation.GO.Term", "Annotation.GO.Category");
  # Write them into a file
  fileName = paste("data_out/", module,"_Transcript_List_Annotation.csv", sep="");
  write.csv(as.data.frame(IDs_ann), file = fileName, row.names = FALSE)
}


#module-trait relationship heat map
#changing column names for aesthetics
names(coldata)[names(coldata)=="Fam84"] <- "Family 84"
names(coldata)[names(coldata)=="Fam89"] <- "Family 89"
names(coldata)[names(coldata)=="Fam90"] <- "Family 90"
names(coldata)[names(coldata)=="Fam120"] <- "Family 120"
names(coldata)[names(coldata)=="Dose2"] <- "Dose"

sizeGrWindow(10,7)

tiff(filename = "figures/Significant_Module-trait_relationships_short_withCor2_newName.tiff",units = "in", width = 6.7 ,height = 6.3, res=300)
# Will display correlations and their p-values
textMatrix <- ifelse(moduleTraitPvalue<=0.05 & moduleTraitPvalue>=0.01, "*",
                  ifelse(moduleTraitPvalue<=0.01 & moduleTraitPvalue>=0.001, "**",
                        ifelse(moduleTraitPvalue<=0.001,  paste(signif(moduleTraitCor, 2), "\n***", sep=""),"")));
dim(textMatrix) = dim(moduleTraitCor)
par(mar = c(6, 8.8, 3, 2.2));
labeledHeatmap(Matrix = moduleTraitCor,
             xLabels = names(coldata),
             yLabels = names(MEs),
             ySymbols = names(MEs),
```

```
            colorLabels = FALSE,
            colors = blueWhiteRed(50),
            textMatrix = textMatrix,
            setStdMargins = FALSE,
            cex.text = 0.5,
            zlim = c(-1,1),
            cex.lab = 0.5,)
dev.off()

#To format data for MWU test, create table for each module with the calculated KME value for transcripts included in module and a KME value of "0" for transcripts not included.

#magenta example: make a new column in the dataframe made above using the ifelse function
TranscriptInfo_ann$magenta <- ifelse(TranscriptInfo_ann$moduleColor == "magenta", TranscriptInfo_ann$kMEmagenta, 0)

#select transcripts and the new column made, which only contains KME values for transcripts in the magenta module
magenta <- TranscriptInfo_ann %>% select("XP", "magenta")
write.table(magenta, "GO_MWU_WGCNA/magenta.csv",
          col.names =TRUE, sep=",", row.names = FALSE, quote=FALSE)

#################################################################
#3) MWU test for WGCNA magenta module biological process ####

#The following code and most comments are from https://github.com/z0on/GO_MWU with some modifications
#Please refer to the GO_MWU github for more details on the MWU protocol (https://github.com/z0on/GO_MWU)

# First, set the directory containing scripts and input files. Then edit, mark, and execute the following bits of code, one after another.
setwd("GO_MWU_WGCNA/")
getwd()

#magenta BP ####
input="magenta.csv" #two columns of comma-separated values: gene id, continuous measure of significance. To perform standard GO enrichment analysis based on Fisher's exact
            #test, use binary measure (0 or 1, i.e., either significant or not).
goAnnotations="Oyster_MWU_Annotation.tab" # two-column, tab-delimited, one line per gene, multiple GO terms separated by semicolon. If you have multiple lines per gene,
            #use nrify_GOtable.pl prior to running this script.
goDatabase="go.obo" # download from http://www.geneontology.org/ on 5/5/2020
goDivision="BP" # either MF, or BP, or CC
source("gomwu.functions.R")
```

```
# ------------- Calculating stats
# It might take a few minutes for MF and BP. Do not rerun it if you just want to replot the data with different cutoffs, go straight to gomwuPlot. If you change any of the numeric
#values below, delete the files that were generated in previous runs first.

gomwuStats(input, goDatabase, goAnnotations, goDivision,
	perlPath="C:/Strawberry/perl/bin/perl", # replace with full path to perl executable if it is not in your system's PATH already
	largest=0.1,  # a GO category will not be considered if it contains more than this fraction of the total number of genes
	smallest=5,   # a GO category should contain at least this many genes to be considered
	clusterCutHeight=0.25, # threshold for merging similar (gene-sharing) terms. See README for details.
	# Alternative="g" # by default the MWU test is two-tailed; specify "g" or "l" of you want to test for "greater" or "less" instead.
	Module=TRUE,Alternative="g" # un-remark this if you are analyzing a SIGNED WGCNA module (values: 0 for not in module genes, kME for in-module genes). In the
			#call to gomwuPlot below, specify absValue=0.001 (count number of "good genes" that fall into the module)
	# Module=TRUE # un-remark this if you are analyzing an UNSIGNED WGCNA module
)
# do not continue if the printout shows that no GO terms pass 10% FDR.

# Plotting results
#quartz()
tiff(filename = "figures/magenta_BP.tiff",units = "in", width = 5.1 ,height = 2.1, res=300)
results=gomwuPlot(input,goAnnotations,goDivision,
		#absValue=-log(0.05,10),  # genes with the measure value exceeding this will be counted as "good genes". This setting is for signed log-pvalues. Specify
			#absValue=0.001 if you are doing Fisher's exact test for standard GO enrichment or analyzing a WGCNA module (all non-zero genes = "good genes").
		absValue=0.001, # un-remark this if you are using log2-fold changes
		level1=0.1, # FDR threshold for plotting. Specify level1=1 to plot all GO categories containing genes exceeding the absValue.
		level2=0.05, # FDR cutoff to print in regular (not italic) font.
		level3=0.01, # FDR cutoff to print in large bold font.
		txtsize=1.2,    # decrease to fit more on one page, or increase (after rescaling the plot so the tree fits the text) for better "word cloud" effect
		treeHeight=0.5, # height of the hierarchical clustering tree
		#	colors=c("dodgerblue2","firebrick1","skyblue2","lightcoral") # these are default colors, un-remark and change if needed
)

dev.off()

# manually rescale the plot so the tree matches the text
```

```
#######################################################################
#4) DESeq2 for differential expression analysis with family 90 as reference ####
#I am using family 90 as reference compared to family 84 as an example with notes indicating how to run the additional family comparisons
#Please refer to the Bioconductor guide "Analyzing RNA-seq data with DESeq2" for more details on the DESeq2 protocol
#(https://bioconductor.org/packages/release/bioc/vignettes/DESeq2/inst/doc/DESeq2.html)


#The following is a list of libraries used.

library(tidyverse)
library(dplyr)
library(plyr)
library(readtext)
library("DESeq2")
library(apeglm)

#DESeqDataSet
#cts = count data for all samples (from CLC Workbench "RNASeq analysis"; please see text for details) in matrix format, each row = transcript and each column = sample
#coldata = metadata for all samples


#subset Dose = 10^8 in metadata table and set Family as factor
coldata <- coldata %>% subset(Dose=="1e+08")
coldata$Fam <- as.factor(coldata$Fam)

#keep count data only for samples in metadata table
cts <- cts[, rownames(coldata)]

#make sure we kept the correct number of samples
ncol(cts)

#make sure our data match up, both should be true
#makes sure we have metadata for every sample in the count matrix and vis versa
all(rownames(coldata) %in% colnames(cts))
all(rownames(coldata)==colnames(cts))

#make DESeq dataset
```

```
dds <- DESeqDataSetFromMatrix(countData = cts,
                              colData = coldata,
                              design = ~ Fam)

#Set the reference treatment
dds$Fam <- relevel(dds$Fam, ref = "90")

#Pre-Filtering
keep <- rowSums(counts(dds)) >= 10
dds <- dds[keep,]

#run DESeq2
dds <- DESeq(dds)

#get results and set alpha level
res <- results(dds, alpha = 0.05)

#get list of coefficients
resultsNames(dds)

#apply apeglm shrinkage to 84 vs 90 coefficient
#repeat the steps below for the remaining two coefficients for additional family comparisons
resApe84 <- lfcShrink(dds, coef="Fam_84_vs_90", type="apeglm", res=res)
head (resApe84)
nrow(resApe84)
summary(resApe84)

#reformat apeglm results as data frame
resApe84 <-as.data.frame(resApe84) %>% rownames_to_column("XM")

#select only significant transcripts
resApeSig84 <- subset(resApe84, padj < 0.05)

#export results
write.csv(as.data.frame(resApeSig84), “data_out/DESeq2_ten8_byfamily_Ref90vs84_D7_Apeglm_sigDESeq_markdown_masked.csv”)

#manipulate our DESeq2 output so that the log2 foldchange for transcripts with a significant adjusted p-value are included and all other transcripts receive a value of 0.
```

```
resApeSig84$L2FC_sig <- ifelse(resApeSig84$padj <=0.05,resApeSig84$log2FoldChange , 0)
resApeSig84  <- resApeSig84 %>% dplyr::select("XP", "L2FC_sig")
names(resApeSig84)[names(resApeSig84)=="XP"] <- "Sequence.Name"
resApe[is.na(resApe84)] <- 0
write.table(resApe84, "MWU_DESeq2/Ref90_vs84_L2FC_sig_expressed.csv",
           col.names =TRUE, sep=",", row.names = FALSE, quote=FALSE)


#################################################################
#5) MWU test for differential expression analysis results

###The following code and most comments are from https://github.com/z0on/GO_MWU with some modifications

# First, set the directory containing scripts and input files. Then edit, mark and execute the following bits of code, one after another.
setwd("MWU_DESeq2/")
getwd()

#Ref90 vs 84 BP ####
input="Ref90_vs84_L2FC_sig_expressed.csv" #made from the above step 4. Two columns of comma-separated values: gene id, continuous measure of significance. To perform
             #standard GO enrichment analysis based on Fisher's exact test, use binary measure (0 or 1, i.e., either significant or not).
goAnnotations="Oyster_MWU_Annotation.tab" # two-column, tab-delimited, one line per gene, multiple GO terms separated by semicolon. If you have multiple lines per gene,
             #use nrify_GOtable.pl prior to running this script.
goDatabase="go.obo" # download from http://www.geneontology.org/ on 5/5/2020
goDivision="BP" # either MF, or BP, or CC
source("gomwu.functions.R")

# Calculating stats. It might take ~3 min for MF and BP. Do not rerun it if you just want to replot the data with different cutoffs, go straight to gomwuPlot. If you change any of the
#numeric values below, delete the files that were generated in previous runs first.
gomwuStats(input, goDatabase, goAnnotations, goDivision,
         perlPath="C:/Strawberry/perl/bin/perl", # replace with full path to perl executable if it is not in your system's PATH already
         largest=0.1,  # a GO category will not be considered if it contains more than this fraction of the total number of genes
         smallest=5,   # a GO category should contain at least this many genes to be considered
         clusterCutHeight=0.25, # threshold for merging similar (gene-sharing) terms. See README for details.
         # Alternative="g" # by default the MWU test is two-tailed; specify "g" or "l" of you want to test for "greater" or "less" instead.
         # Module=TRUE,Alternative="g" # un-remark this if you are analyzing a SIGNED WGCNA module (values: 0 for not in module genes, kME for in-module genes). In the
                  #call to gomwuPlot below, specify absValue=0.001 (count number of "good genes" that fall into the module)
         # Module=TRUE # un-remark this if you are analyzing an UNSIGNED WGCNA module
```

```
)
# do not continue if the printout shows that no GO terms pass 10% FDR.

)
# Plotting results
#quartz()
#dev.new()
tiff(filename = "figures/Ref90_vs84_L2FC_sig_expressed_BP.tiff",units = "in", width = 5.4, height = 1.7, res=300)
results=gomwuPlot(input,goAnnotations,goDivision,
		#absValue=-log(0.05,10),  # genes with the measure value exceeding this will be counted as "good genes". This setting is for signed log-pvalues. Specify
			#absValue=0.001 if you are doing Fisher's exact test for standard GO enrichment or analyzing a WGCNA module (all non-zero genes = "good genes").
		absValue=0.001, # un-remark this if you are using log2-fold changes (Note from authors: changed to include all transcripts with a non-zero L2FC as "good")
		level1=0.1, # FDR threshold for plotting. Specify level1=1 to plot all GO categories containing genes exceeding the absValue.
		level2=0.05, # FDR cutoff to print in regular (not italic) font.
		level3=0.01, # FDR cutoff to print in large bold font.
		txtsize=1.0,    # decrease to fit more on one page, or increase (after rescaling the plot so the tree fits the text) for better "word cloud" effect
		treeHeight=0.5, # height of the hierarchical clustering tree
		#	colors=c("dodgerblue2","firebrick1","skyblue2","lightcoral") # these are default colors, un-remark and change if needed
)
#dev.off()

# manually rescale the plot so the tree matches the text


##################################################################
#6) Venn diagram ####

#For the following script I used the R package "VennDiagram".  Please see documentation for more information (https://cran.r-
#project.org/web/packages/VennDiagram/VennDiagram.pdf)

#The following is a list of libraries used.

library(VennDiagram)
library(RColorBrewer)

#from DESeq2 results for each family, see above step 4.
```

```
fam84_string <- unlist(resApeSig84)
fam89_string <- unlist(resApeSig89)
fam120_string <- unlist(resApeSig120)


#create Venn diagram
venn.diagram(x = list(
  "Family 84" = fam84_string,
  "Family 89" = fam89_string,
  "Family 120" = fam120_string), filename = "figures/Ten8_byFamily_ref90_Sig_masked_2.png",
  fill = c("black", "red", "purple"),
  alpha = 0.4,
  col = "transparent",
  cex = 1.5,
  cat.col = c("black", "black", "black"),
  fontfamily = "serif",
  fontface = "bold",
  cat.dist = 0.08,
  cat.cex = 1.5,
  cat.fontfamily = "serif",
  margin = 0.2
);

#Get lists of overlapping transcripts
overlap <- calculate.overlap(list(
  "84" = fam84_string,
  "89" = fam89_string,
  "120" = fam120_string))

#re-name lists so they make sense
names(overlap) <- c("a123", "a12", "a13", "a23", "a1", "a2", "a3") # 1 = 84, 2= 89, 3= 120 as designated in the list for "calculate.overlap"

#get everything in environment
list2env(overlap,envir=.GlobalEnv)

#export for downstream analyses example: transcripts differentially expressed in DESEq2 comparison for both 84/90 and 89/90:
```

```
#convert to a tibble
a12 <- as_tibble(a12)

#create txt file for OmicsBox
write.table(a12, file= "data_out/VennOverlap_ten8_byfamily_Ref90vs84_Ref90vs89_Sig_masked.txt",
            row.names=FALSE, col.names=FALSE, sep = "\t", quote = FALSE)

#reformat and merge with annotation file, please see text for information on how B2G annotation file was created
names(a12)[names(a12)=="value"] <- "XP"
a12 <- merge(a12, ann_XP_XM, by="XP")
write.csv(a12, file= "data_out/VennOverlap_ten8_byfamily_Ref90vs84_Ref90vs89_Sig_masked.csv")
```
