# Supplementary material for: Understanding Crassostrea virginica tolerance of Perkinsus marinus through global gene expression analysis
Source: Front Genet. 2023 Jan 19;14:1054558. doi: 10.3389/fgene.2023.1054558 (PMC9892467; doi:10.3389/fgene.2023.1054558)

Supplemental 2

Hierarchical clustering trees created from Fisher-test mode for adaptive clustering combined with a Mann-Whitney U (MWU) test of weighted gene correlation network analysis (WGCNA) significant modules.

Black: biological process (BP)
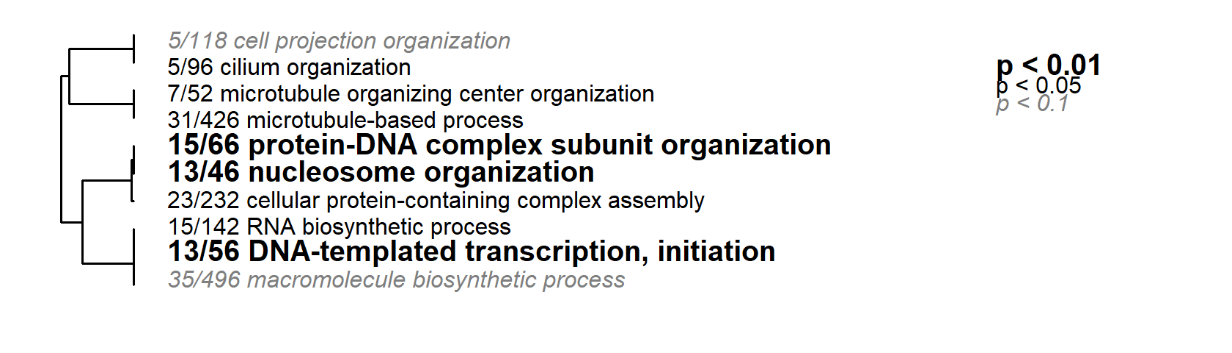


Black: cellular component (CC)


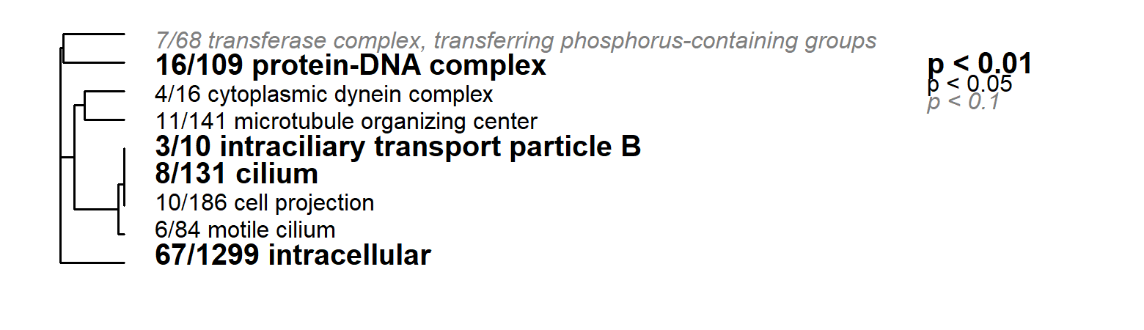


Black: molecular function (MF)


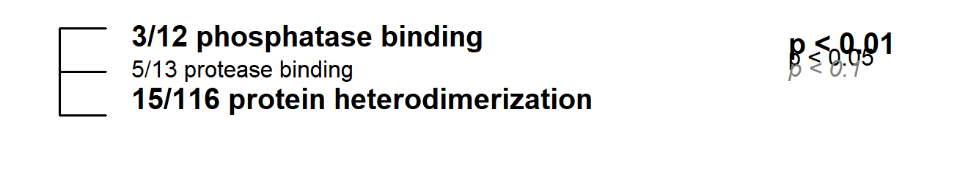


Blue: BP


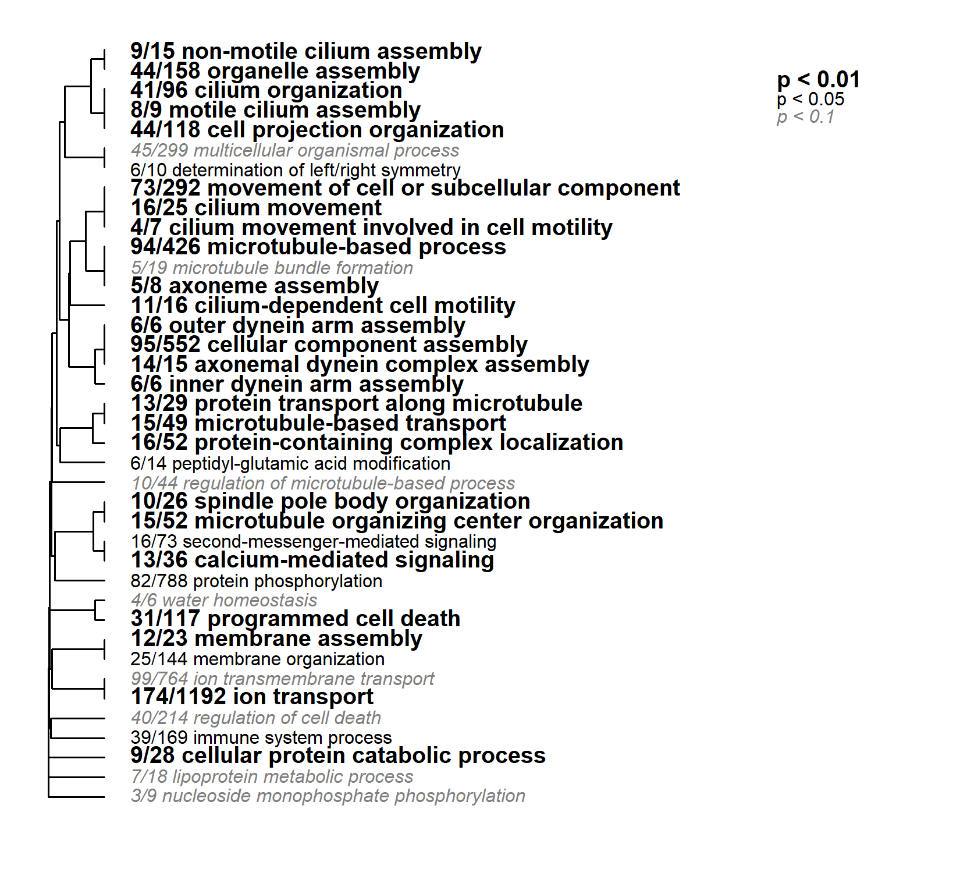


Blue: CC


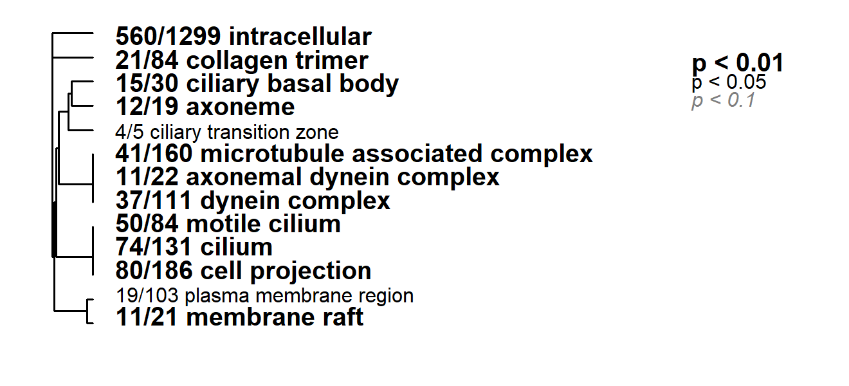


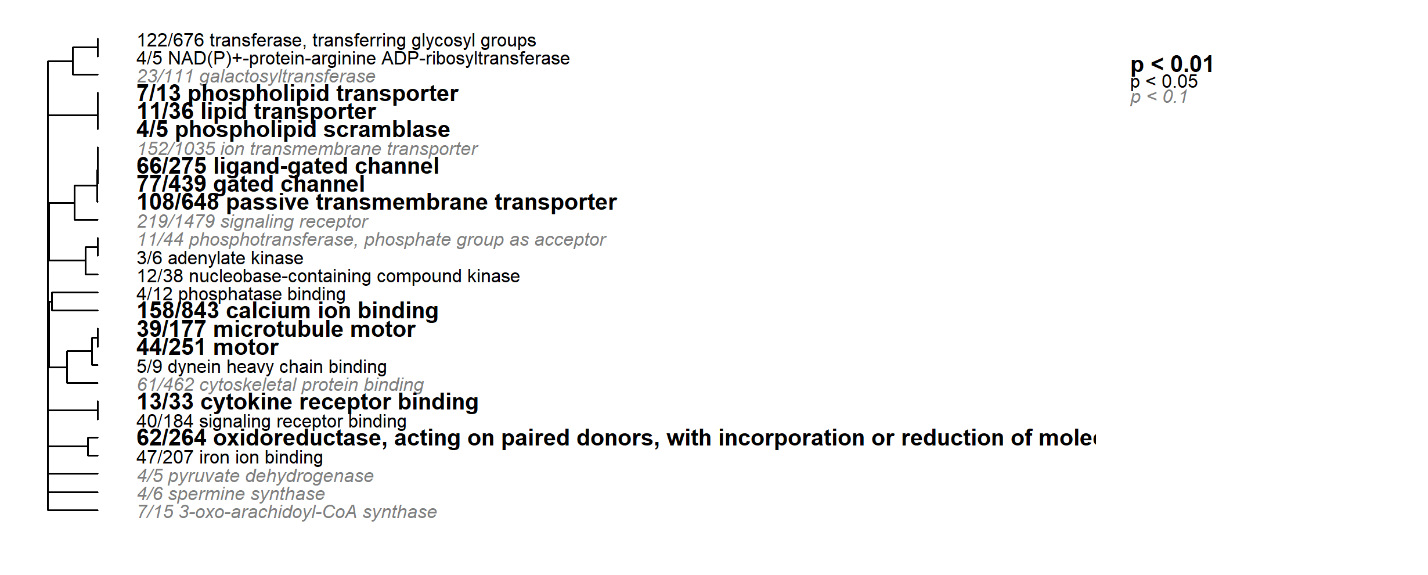
Blue: MF


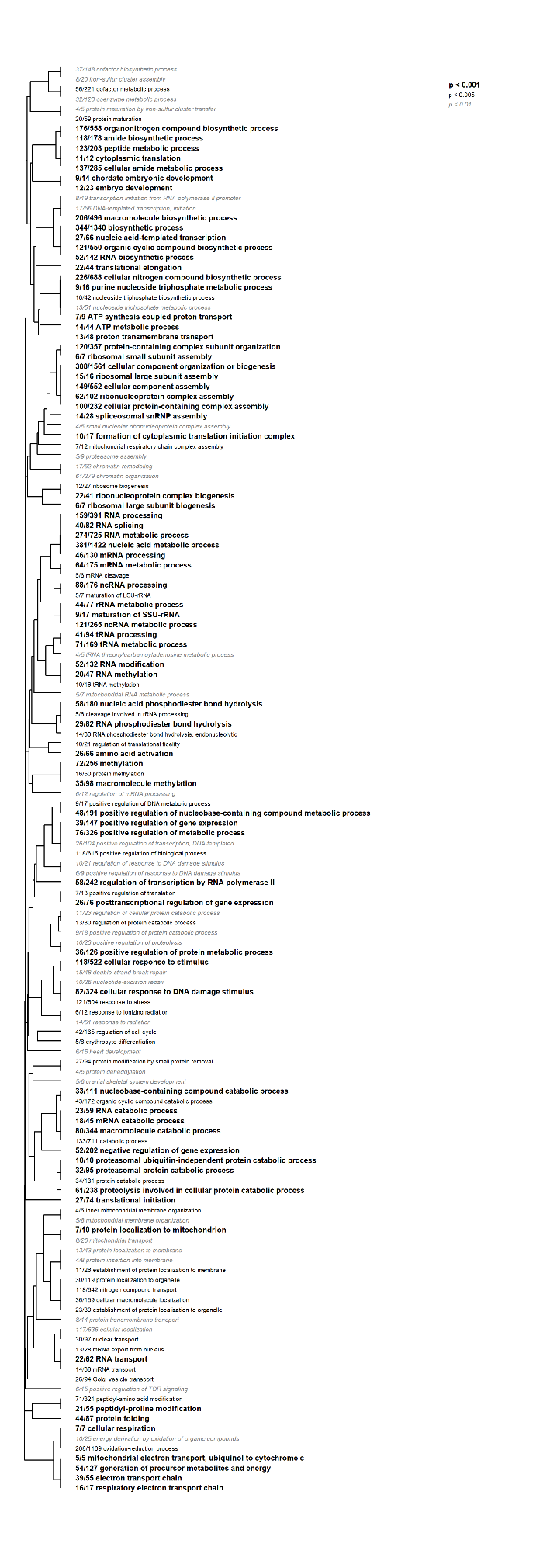
Brown: BP (P-value cut off 0.01)

Brown: CC


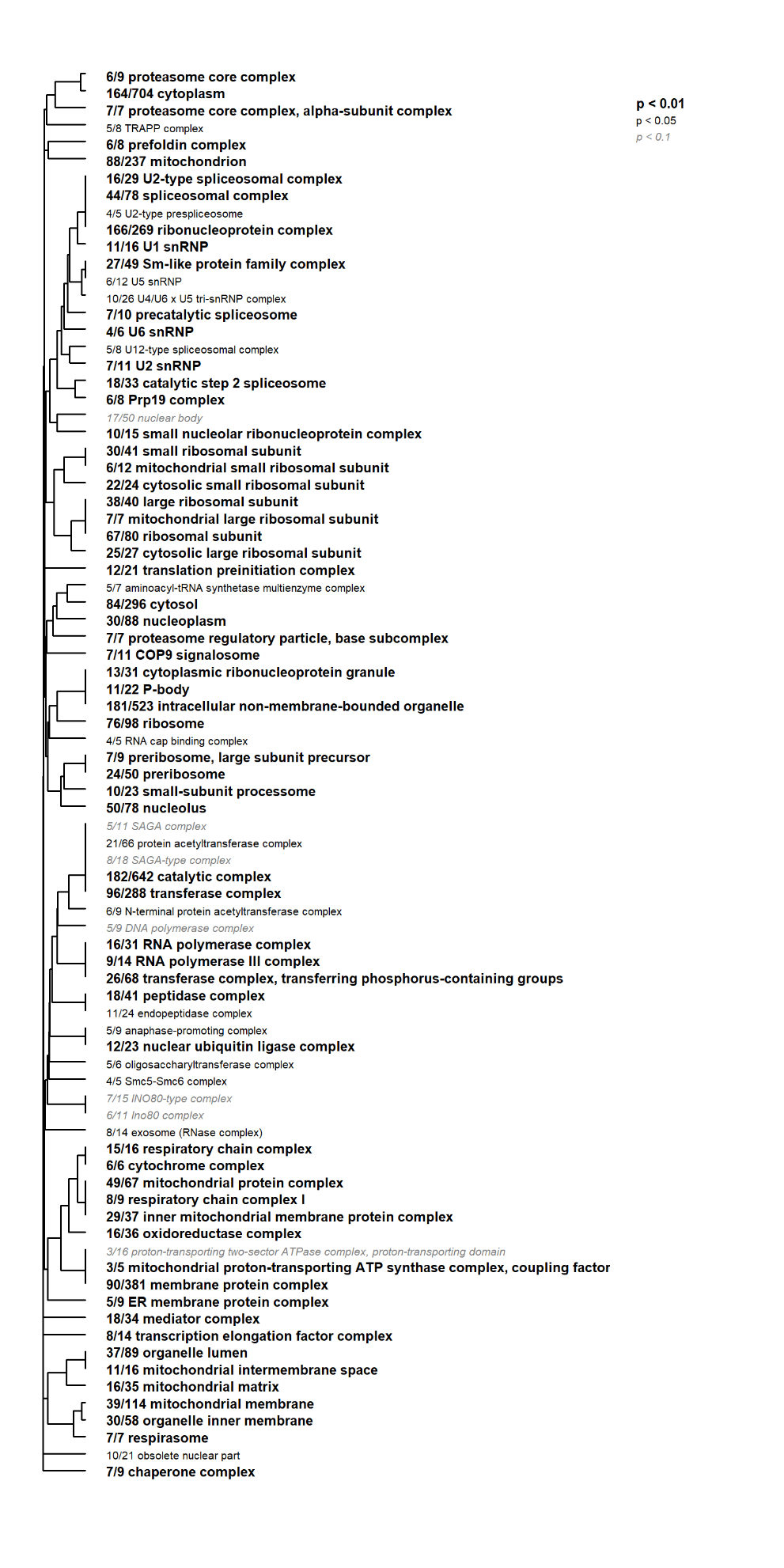


Brown: MF


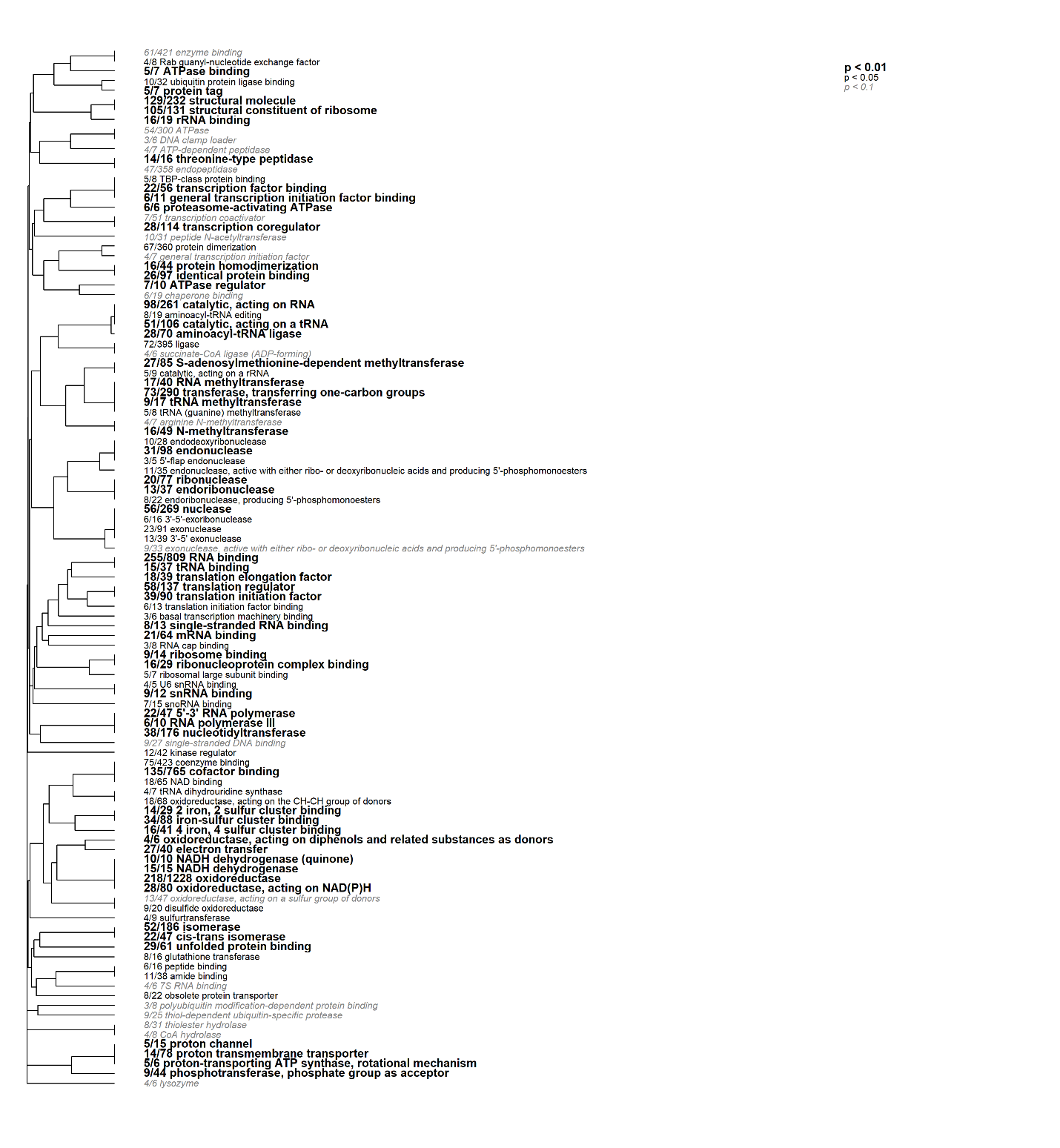


Cyan: BP


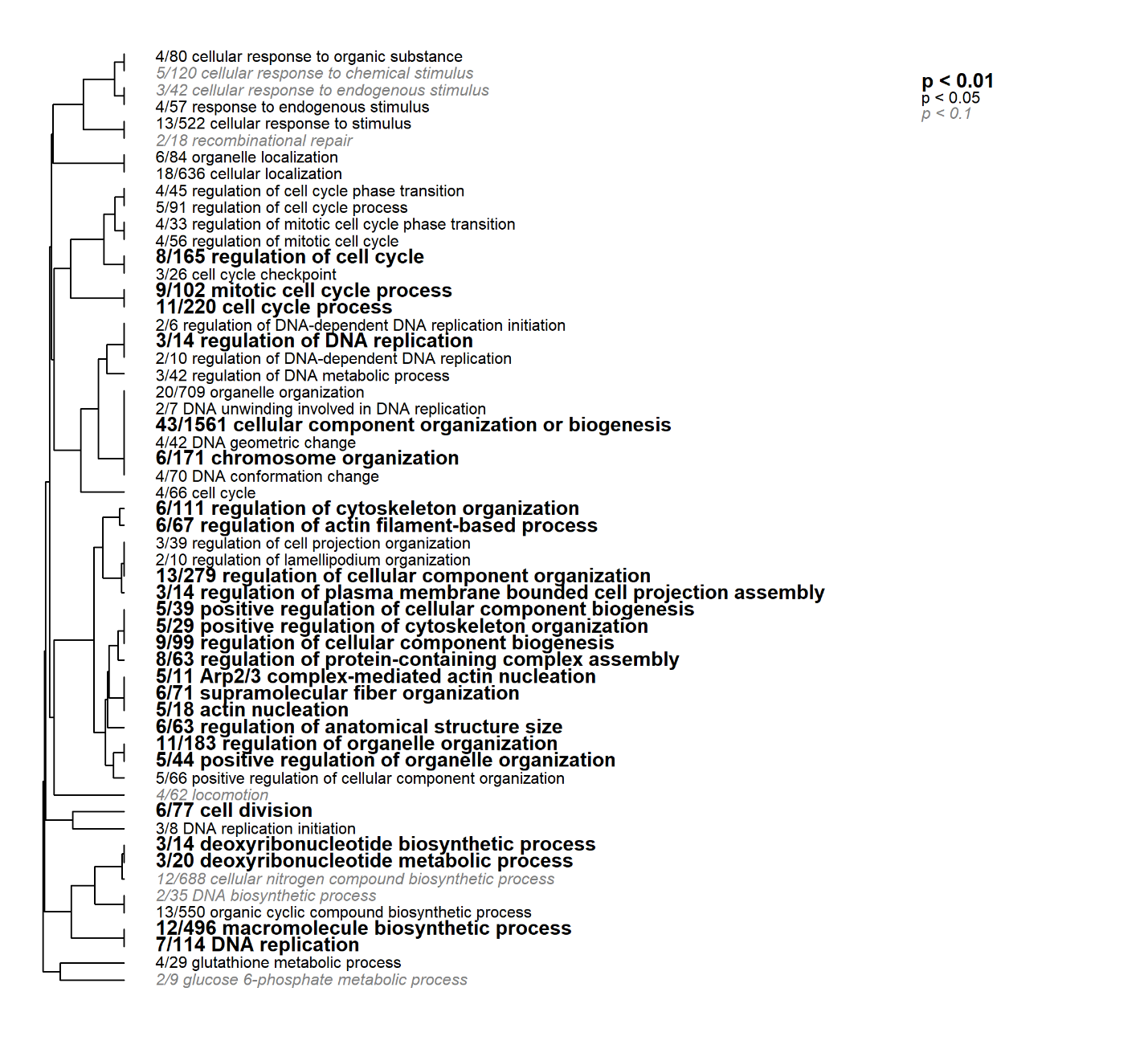


Cyan: CC


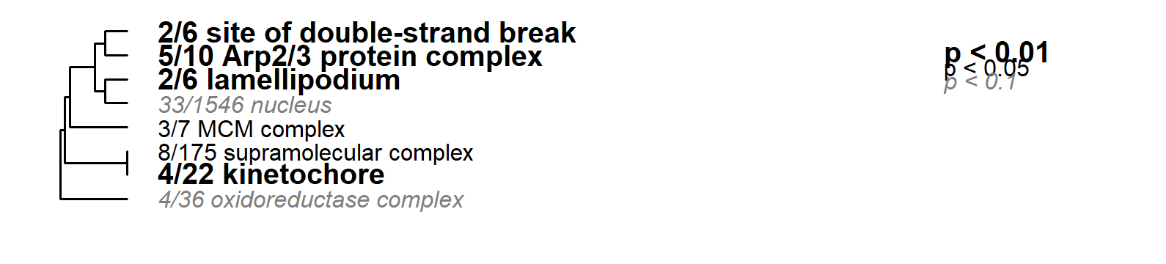


Cyan: MF


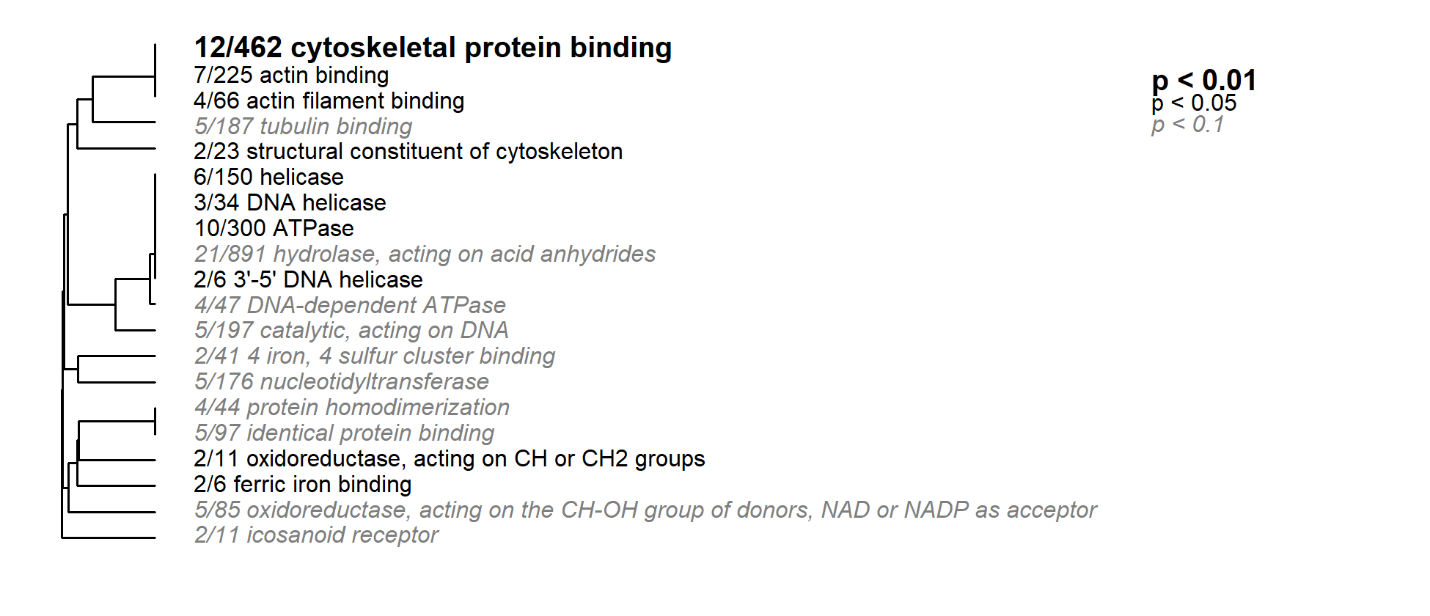


Green: BP


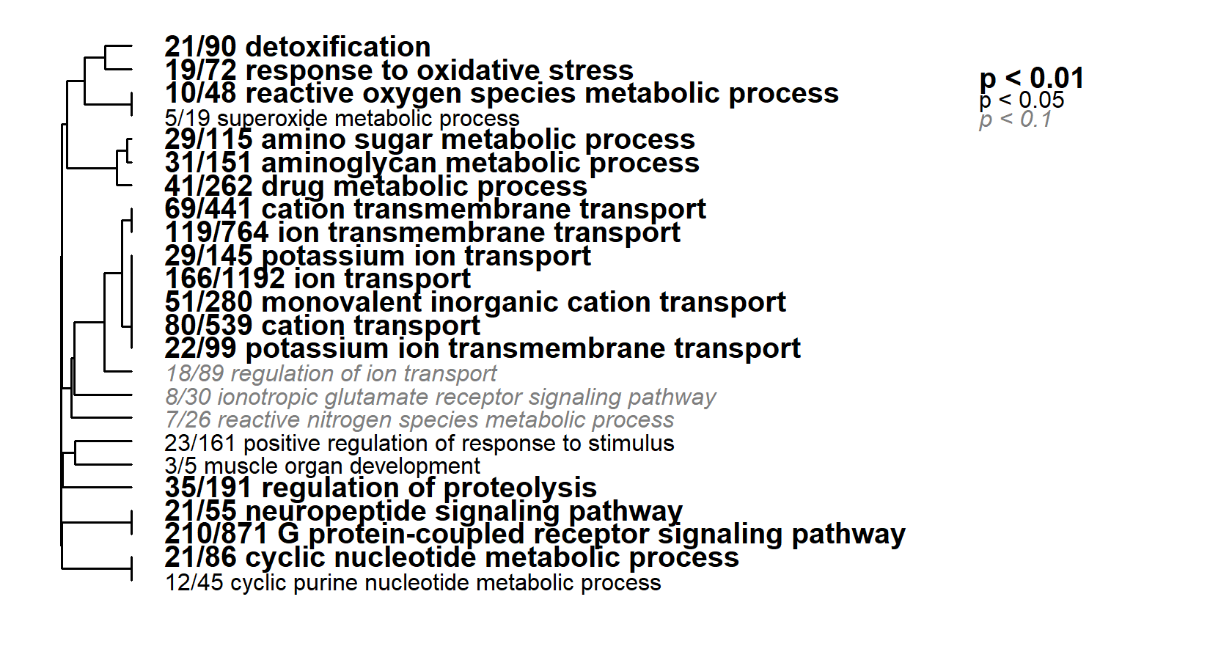


Green: CC


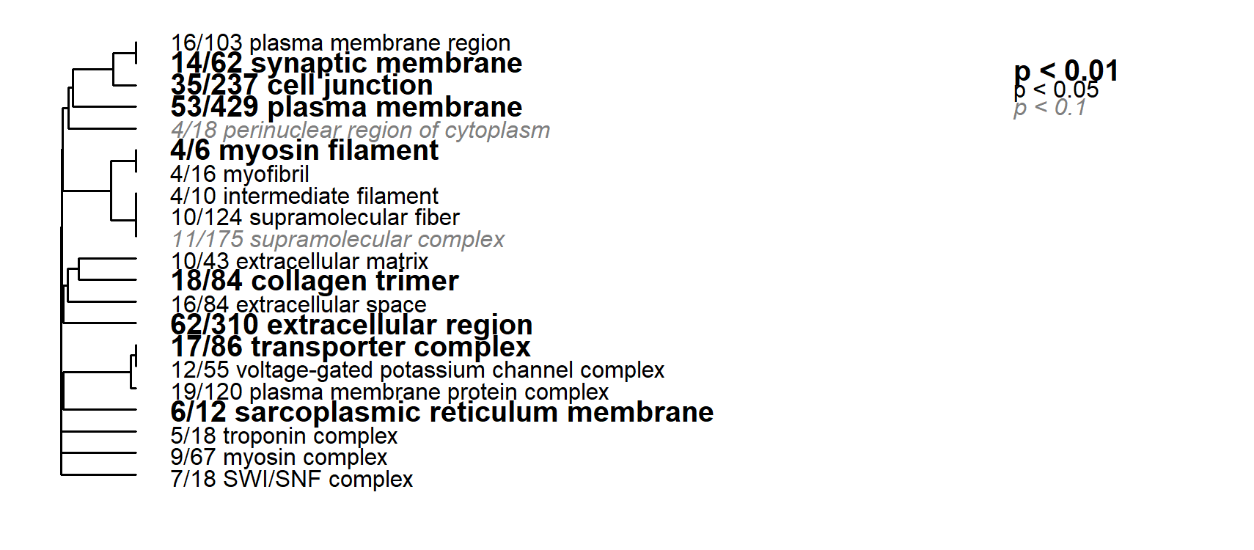


Green: MF


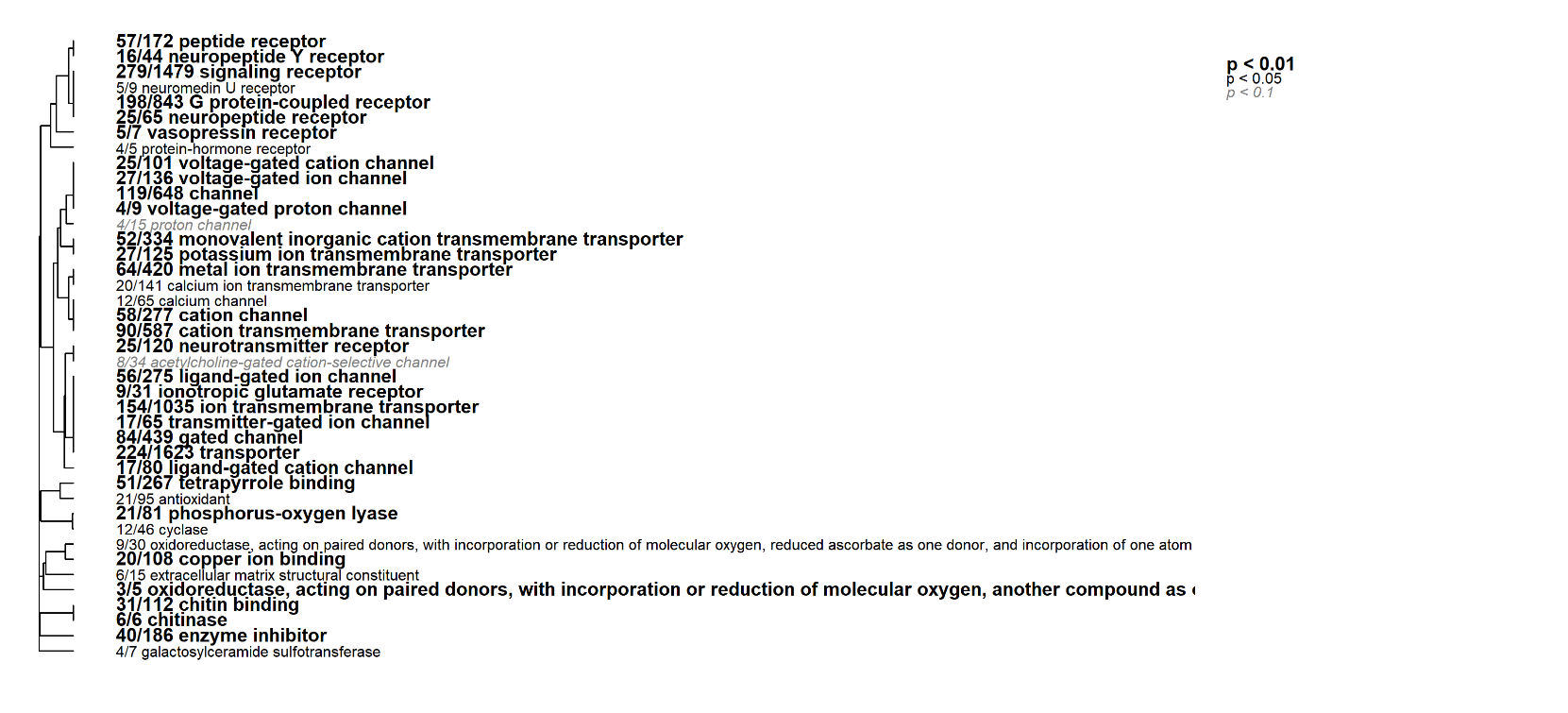


Greenyellow: BP


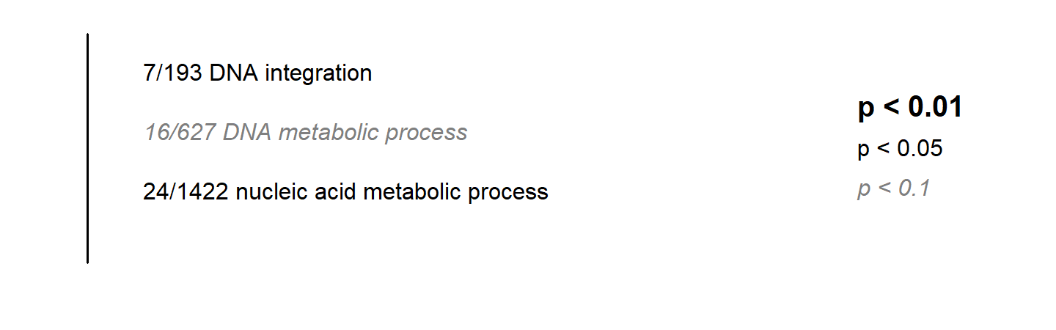


Magenta: BP


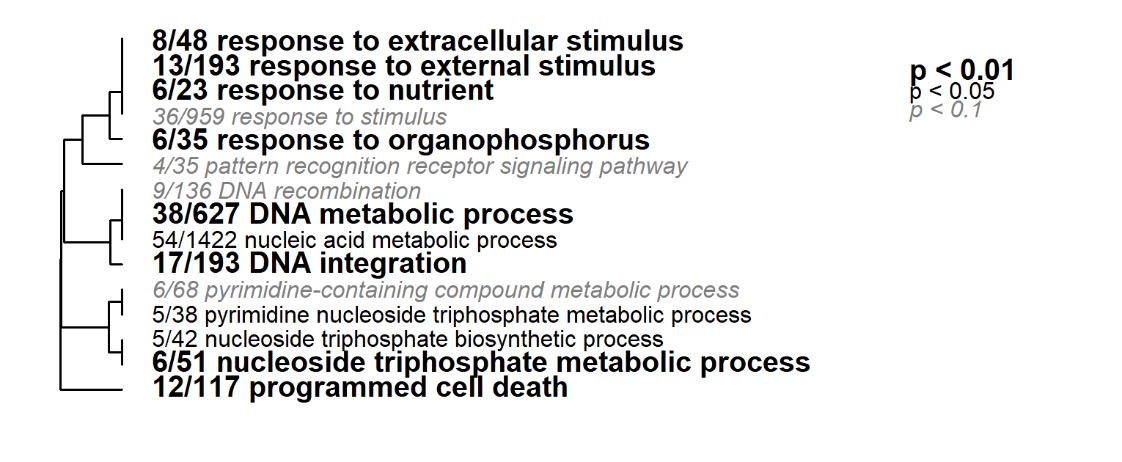


Magenta: MF


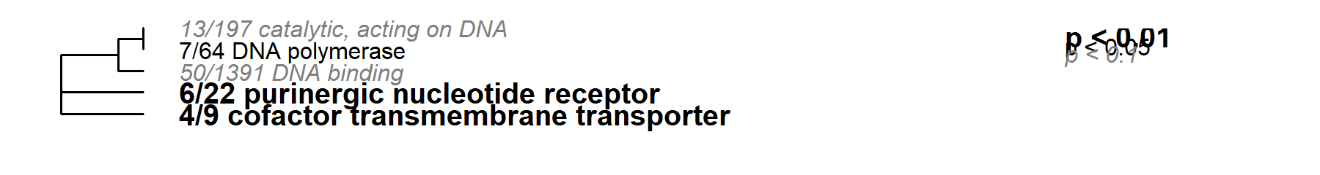


Midnightblue: BP


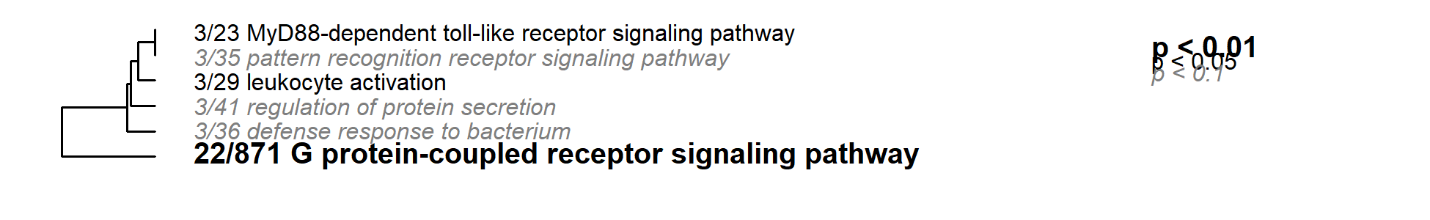


Midnightblue: CC


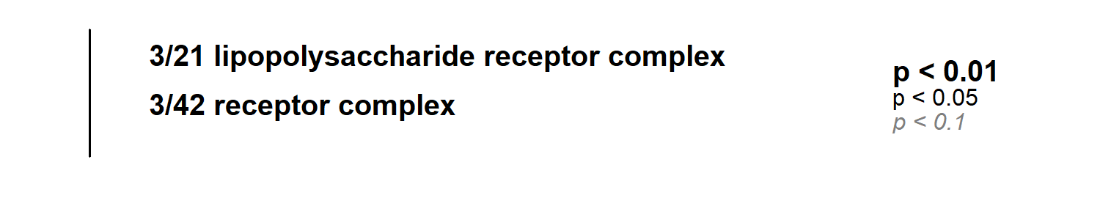


Midnightblue: MF


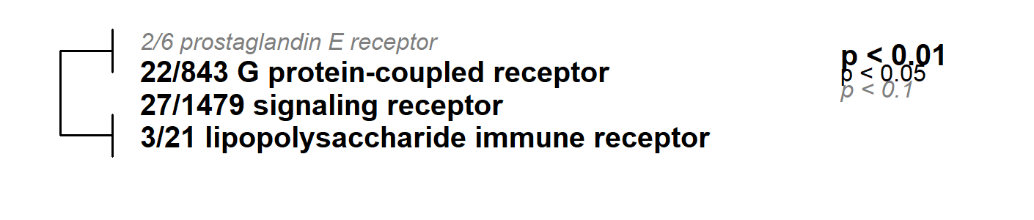


Pink: BP


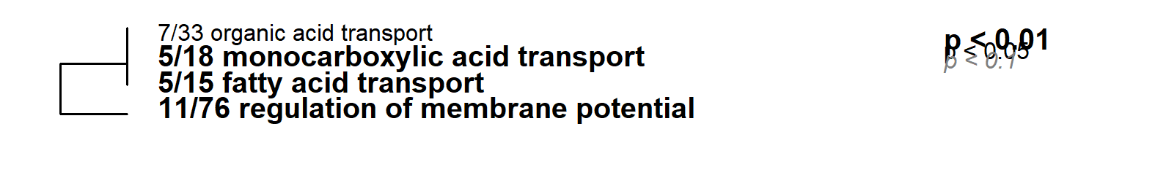


Pink: MF


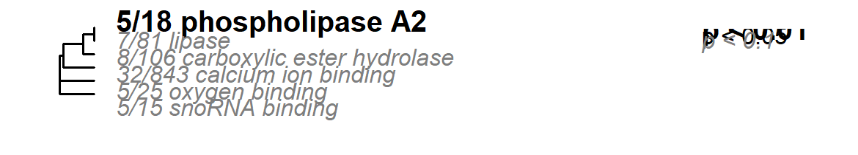


Purple: BP


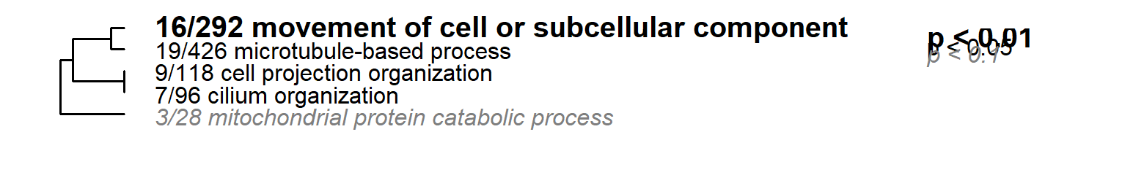


Purple: CC


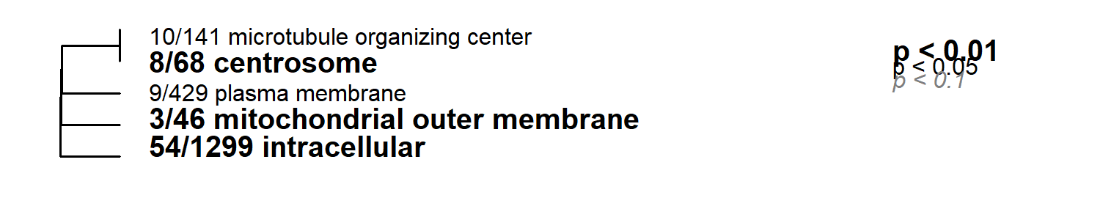


Purple: MF


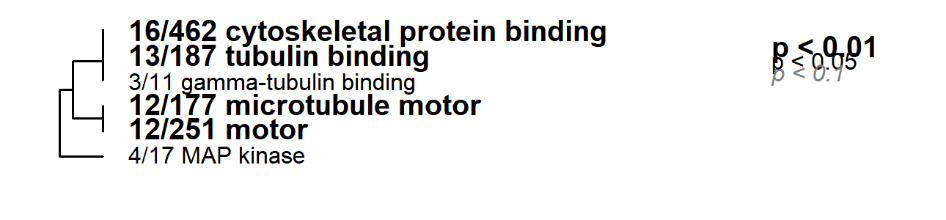


Salmon: BP


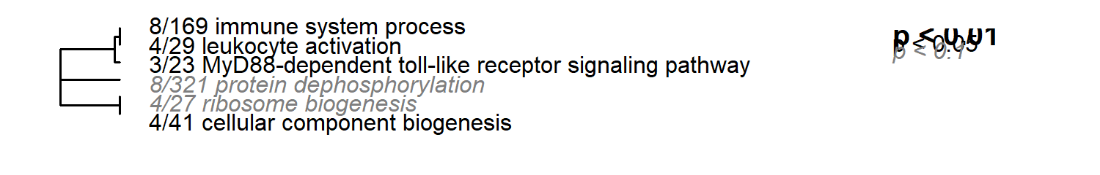


Salmon: MF


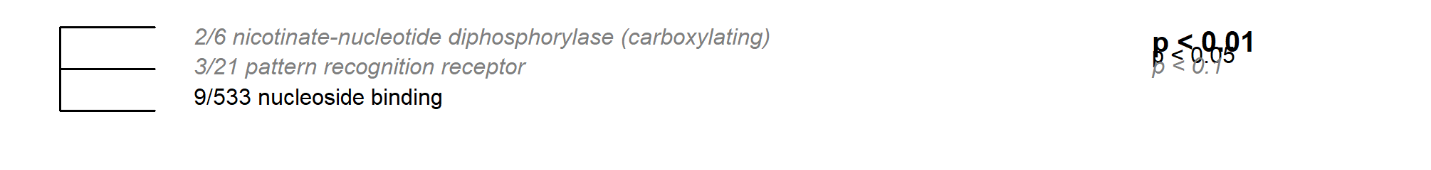


Turquoise: BP (P-value cut off 0.01)


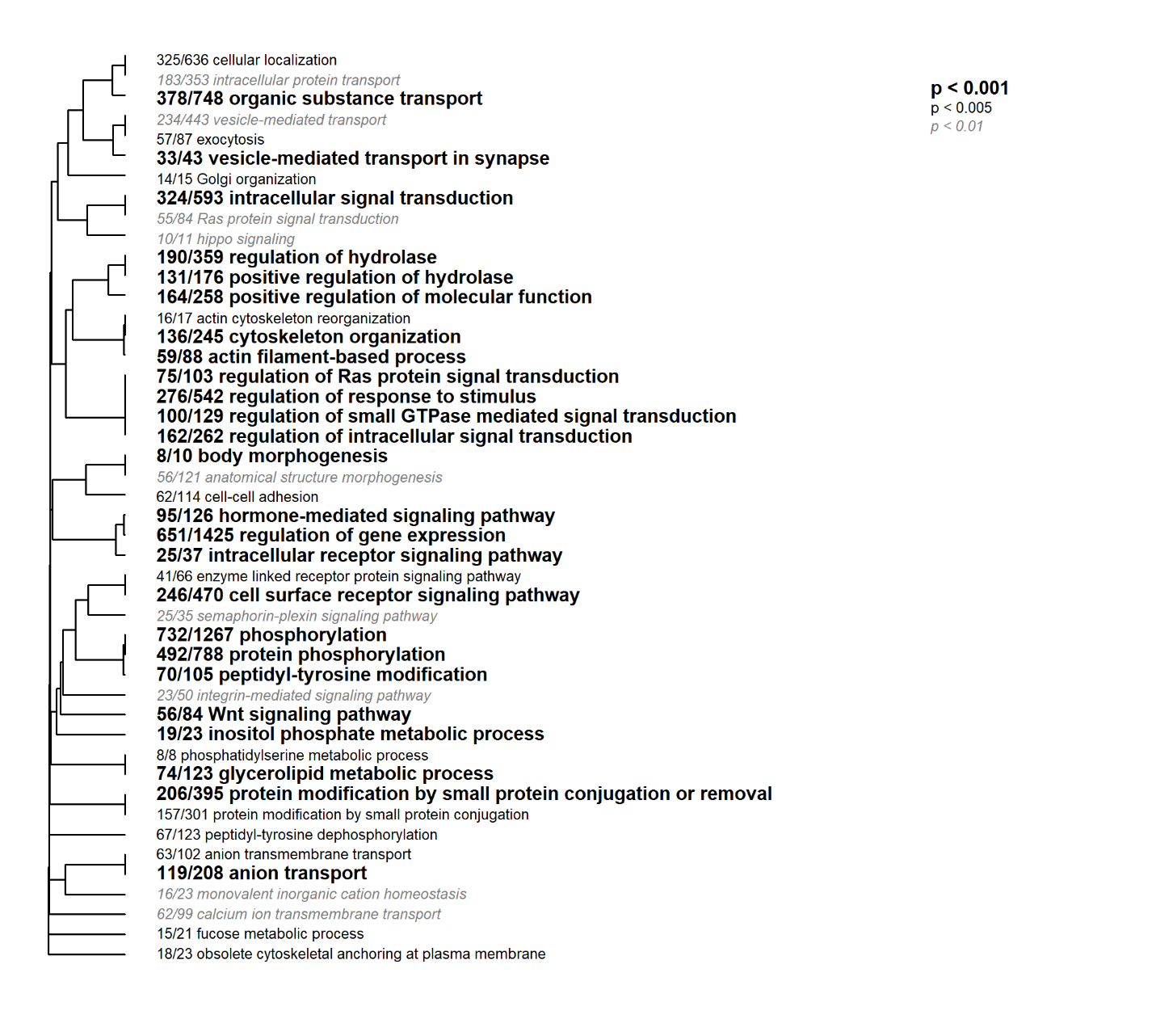


Turquoise: CC


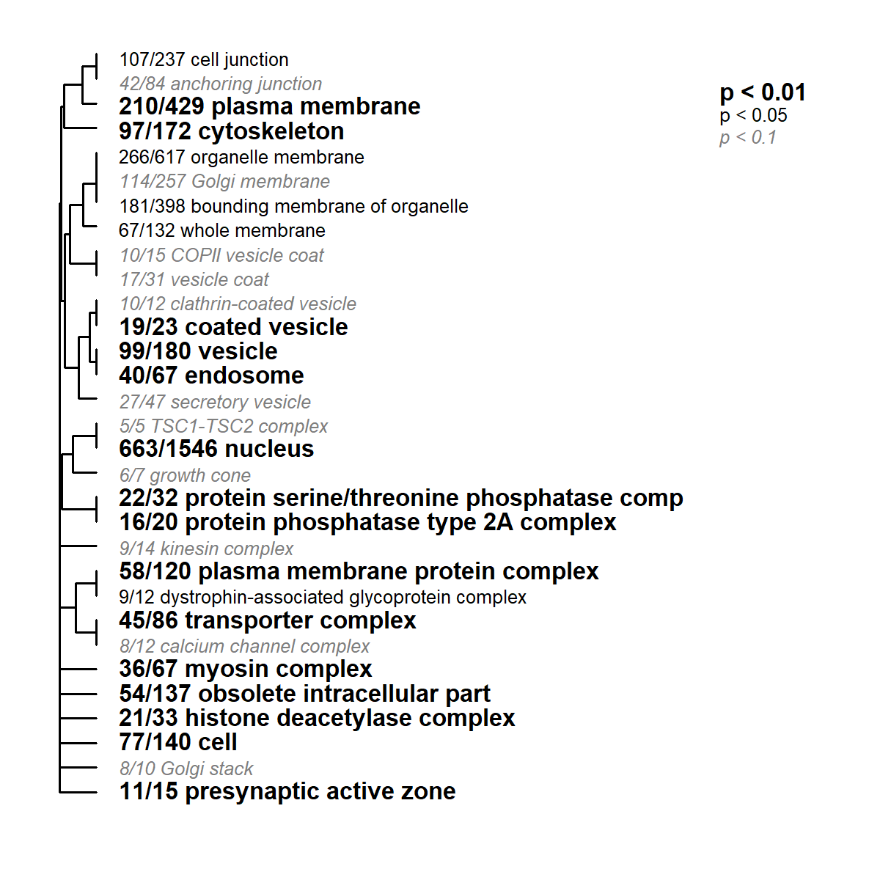


Turquoise: MF (P-value cut off 0.01)


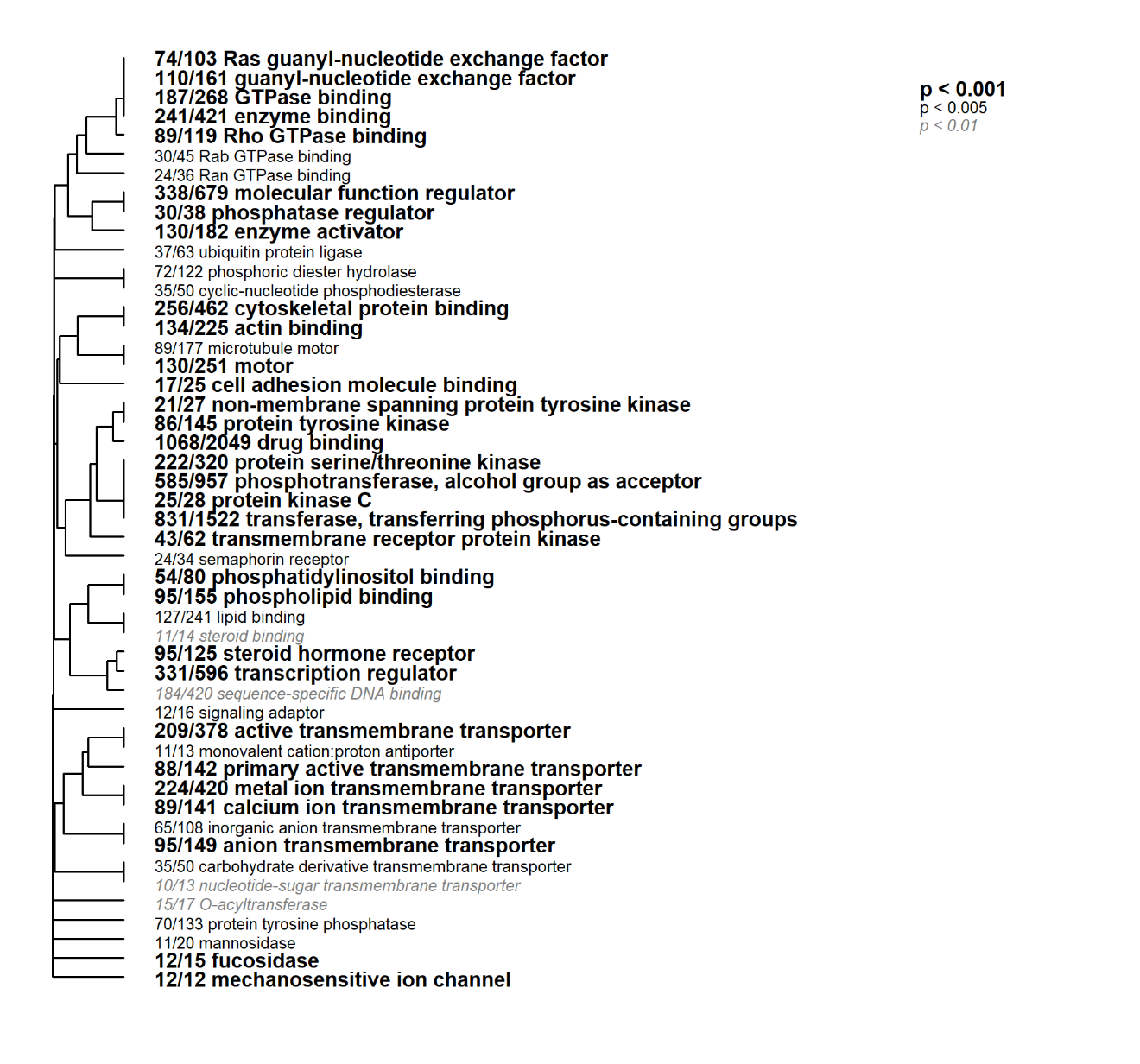

Supplement: Supplementary file 6 [file DataSheet2.docx]
